# Machine learning methods for predicting human-adaptive influenza A virus reassortment based on intersegment constraint

**DOI:** 10.3389/fmicb.2025.1546536

**Published:** 2025-03-21

**Authors:** Dan-Dan Zeng, Yu-Rong Cai, Sen Zhang, Fang Yan, Tao Jiang, Jing Li

**Affiliations:** ^1^College of Veterinary Medicine, Shanxi Agricultural University, Jinzhong, China; ^2^State Key Laboratory of Pathogen and Biosecurity, Academy of Military Medical Sciences, Beijing, China

**Keywords:** influenza A viruses (IAVs), nucleotide composition, reassortment, machine learning, H1N1

## Abstract

**Introduction:**

It is not clear about mechanisms underlining the inter-segment reassortment of Influenza A viruses (IAVs).We analyzed the viral nucleotide composition (NC) in coding sequences,examined the intersegment NC correlation, and predicted the IAV reassortment using machine learning (ML) approaches based on viral NC features.

**Methods:**

Unsupervised ML methods were used to examine the NC difference between human-adapted and zoonotic IAVs. Supervised ML models of random forest classifier (rfc) and multiple-layer preceptor (mlp) were developed to predict the human adaption to IAVs.

**Results:**

Our results demonstrated that the frequencies of thymine, cytosine, adenine,and guanine (t, c, a, and g), as well as the content of gc/at were consistently high or low for the segments of *PB2*, *PB1*, *PA*, *NP*, *M1*, and *NS1* (ribonucleoprotein plus [RNPplus]), between mammalian and avian IAVs or between influenza B viruses (IBVs) and IAVs.RNPplus NC negatively correlated with the NC for *HA*, *NA*, and *M1* (envelope protein plus [EPplus]). The human-adapted NC accurately discriminated between human IAVs and avian IAVs. A total of 221,184 simulated IAVs with pd09H1N1 EPplus and with RNPplus from other IAV subtypes indicated a high adaption of the RNPplus, from H6N6, H13N2, and H13N8 and other IAVs.

**Discussion:**

In summary, there is a distinct human adaption-specific genomic NC between human IAVs and avian IAVs. The intersegment NC correlation constrains segment reassortment. This study presents a novel strategy for predicting IAV reassortment based on viral genetic compatibility.

## Highlights

- There was a correlation between the intersegment nucleotide composition and the genomic nucleotide composition of influenza A viruses (IAVs).
- A machine-learning (ML) approach, based on features of viral nucleotide composition, predicted adaptive IAV reassortment.
- The H6N6, H13N2, and H13N8 IAVs exhibited a high degree of human adaptation when their ribonucleoprotein plus (RNPplus), comprising segments of *PB2*, *PB1*, *PA*, *NP*, and *NS1*, was simulated and recombined with pd09H1N1 the envelope protein plus (EPplus), which includes segments of *HA*, *NA*, and *M1*.

## 1 Introduction

Influenza A viruses (IAVs) are negative-sense, single-stranded, segmented RNA viruses, the genomes that contain eight RNA segments comprising more than 13,000 bases (Eisfeld et al., 2015; Te and Fodor, 2016). IAVs lack a proofreading function in RNA polymerase, resulting in a high mutation rate of 10^−3^ to 10^−4^ during replication (Ahlquist, 2002; Chen and Holmes, 2006; Liu et al., 2014). Mutations in structurally or functionally significant sites in IAVs (Sun H. et al., 2014; Taubenberger and Kash, 2010; Webster et al., 1992) drive a rapid virus evolution. Moreover, a high intersegment reassortment provides IAVs with greater evolutionary space and genetic diversification (Mehle et al., 2012), even though IAVs primarily infect avian or mammalian hosts (Deng et al., 2017), generally exhibiting species specificity. It is concerning that another outbreak of the H5N1 IAV, which initially originated in Europe at the end of 2020 (Adlhoch et al., 2022), has subsequently spread throughout the United States (Bevins et al., 2022) and has resulted in widespread infections in mammals across Europe (Adlhoch et al., 2022) and North America (Elsmo et al., 2023), as well as in the countries of Peru and Chile in Central and South America (Leguia et al., 2023; Sevilla et al., 2024; Castro-Sanguinetti et al., 2024). Thus, there is a high reassortment risk of the prevalent H5N1 and H6N2 viruses (Abolnik, 2024), as well as human IAVs. This risk is based on the fact that five of the last recorded influenza pandemics were caused by avian- or swine-origin or reassorted IAVs (Bragstad et al., 2011; Long et al., 2019; Reid et al., 2004; Kislinger et al., 2006). However, the mechanisms underlying adaptive IAV reassortment are unknown.

Interestingly, it seems that IAV segments do not reassort randomly with each other; in other words, there is a frequency bias for all eight segments in the reassortment (Marshall et al., 2013) due to multiple factors (Lowen, 2017). This bias is observed in the field and has been confirmed experimentally (Arai et al., 2019; Chen et al., 2008; Kimble et al., 2011; Octaviani et al., 2010). First, accessibility in space and time is essential for such reassortment. A coinfection of two or more IAVs in the same host or host cell is necessary for virus reassortment (Marshall et al., 2013). Second, the incompatibility of IAV segments among heterologous RNA packaging signals, particularly at both the 5′ and 3′ terminals (non-coding sequences and parts of coding sequences), restricts the reassortment between H3N2 and H5N2 or between H1N1 and H3N2 viruses (Cobbin et al., 2014; Essere et al., 2013; Sun W. et al., 2014). Third, the compatibility among viral proteins, such as polymerase subunit proteins, exists between H7N7 and H3N2 IAVs (Li et al., 2008), as well as between H1N1 and H5N1 viruses (Naffakh et al., 2000). The balance between *HA* avidity and *NA* activity is another crucial compatibility restriction in protein level for IAV reassortment (Naffakh et al., 2000; Wagner et al., 2002). Thus, there is a constraint on intersegment reassortment for IAVs from avian and mammalian hosts.

IAV comprises eight viral RNA segments (*PB2*, *PB1*, *PA*, *HA*, *NP*, *NA*, *M*, and *NS*) and eight structural proteins, all of which are delicately packaged. Regarding the role of viral RNAs in viral packaging, the hydrogen bonds between nucleotides a and t, as well as between nucleotides c and g, contribute to the secondary structure stability of segmented viral RNAs. It is reasonable to infer that the nucleotide composition of viral RNAs may be highly significant for the folding free energy and in the structural stability, both of which regulate IAV evolution and host adaptation (Brower-Sinning et al., 2009). An ordered RNA structure was found in IAV segments of *PB2*, *NP*, *M*, and *NS* that varied in free energies for secondary RNA structure formation among virus strains from avian, swine, and human species (Priore et al., 2012). The nucleotide composition, particularly dinucleotides (Gaunt et al., 2016; Greenbaum et al., 2014), mononucleotides, and tetranucleotides (Iwasaki et al., 2013) in IAV coding sequences, has been indicated to be structurally and functionally crucial for IAVs. Both experimental and computational evidence has demonstrated significant roles of nucleotide composition in regulating host innate immune response (Takata et al., 2017), virulence (Atkinson et al., 2014; Tulloch et al., 2014), and viral replication (Witteveldt et al., 2016). Therefore, we hypothesized that a type of genetic compatibility for IAV reassortment may exist based on viral nucleotide composition.

In this study, we analyzed the viral nucleotide composition by counting the frequency of the four types of nucleotides, t, c, a, and g, calculating the gc and at content, the theoretical gc or/and at pairs, and the pair-free nucleotide in the full-length coding sequences for each of the eight IAV segments. The importance and differences in each of these nucleotide factors were analyzed using machine learning (ML) methods, and the intersegment correlation of these factors was also assessed with Pearson correlation. Subsequently, we simulated reassortant IAVs with pandemic (H1N1) 2009 viruses (pd09H1N1) and other IAVs before 2009. This study presents a novel strategy for predicting IAV reassortment based on viral genetic compatibility.

## 2 Materials and methods

### 2.1 Sequence data processing

A total of 442,893 coding sequences from all eight IAV segments, including polymerase basic protein 2 (*PB2*), polymerase basic protein 1 (*PB1*), polymerase acidic protein (*PA*), hemagglutinin (*HA*), nucleoprotein (*NP*), neuraminidase (*NA*), matrix protein 1 (*M1*), and nonstructural protein 1 (*NS1*), were downloaded from the Influenza Research Database (IRD) and from Global Initiative on Sharing All Influenza Data (GISAID) database (Shu and McCauley, 2017), as of 31 December 2018. Full-length sequence samples were utilized for strain genome assembly and nucleotide composition analysis. IAVs had all the eight segmental full sequences assembled in order (first to eighth: *PB2*, *PB1*, *PA*, *HA*, *NP*, *NA*, *M1*, and *NS1*), creating a complete viral genomic coding sequence (*n* = 12, 400, within which 11, 861 samples from IRD and the remainder from GISAID). A stochastic resampling was performed to reduce the distribution bias on the USA of the Country_area label, and on the 2009 of the Year label, 9,525 strains of IAVs were left after dropping out 2,336 samples.

### 2.2 Counting of genomic nucleotide composition

A counting script was designed for analyzing NC features for each IAV gene sample based on previously reported methods (Jiang et al., 2023; Li et al., 2023, 2020; Zhang et al., 2024). The algorithms were designed according to Equations 1, 2, respectively. Statistical descriptions of variants were performed based on sample annotation information. The full-coding DNA sequence (CDS) for each sample with unknown nucleotide less than 1% was analyzed for its frequency of nucleotide (nt, t, c, a, and g), dinucleotide (dnt, tt, tc, ta, tg, ct, cc, ca, cg, at, ac, aa, ag, gt, gc, ga, and gg based on the position of first, second, and third for the first nt in a codon) and amino acid (aa). A vector with a dimension of 12, 48, or 20 was produced for nt, dnt, or aa, respectively.


(1) $$freqx=\frac{\Sigma x}{\Sigma_{i=1}^{4}x},x=t,c,a\;\mathrm{or}\;g$$

(2) freqxnym=ΣxnymΣi=116xnym,(x,y=t,c,aorg,m=n+1form≤3,m=n−2form=4,n=codonntposition1,2,or3

Codon-pair features of the reassorted genome of simulated IAVs were also analyzed based on the previously reported tool ().

### 2.3 IAV simulation with EPplus of pd09H1N1 IAVs and RNPplus of non-H1N1 IAVs

The reassortment of pd09H1N1 IAVs with other IAVs was performed with a Python script (https://github.com/Jamalijama/IAVreassormentConstraint). The reassortment between the pd09H1N1 virus and different subtypes of IAVs was simulated with the segments of *HA*, *NA*, and *M1* from 36 pandemic human-originated H1N1 strains isolated in 2009 in the USA, and with the segments of *PB2*, *PB1*, *PA*, *NP*, and *NS1* from 6,144 non-H1N1 IAVs, from various host types. The nucleotide composition features for these simulated viruses were counted with the above-mentioned methods.

### 2.4 Unsupervised and supervised machine learning

The ML analysis was performed with the Scikit-learn package (version = 0.18.1, https://scikit-learn.org/stable/#, Python language) or Scipy package (cluster.hierarchy, version = 0.19.0, https://www.scipy.org). “Sklearn.decomposition.PCA” was utilized for principal component analysis (PCA) (Jolliffe and Cadima, 2016), with which nucleotide composition features for multiple segments were reduced into one principal component, with the most significant possible variance (Equation 3). The separability in NC or codon-pair features between human and avian IAVs was assessed by feature reduction and pairplot. Another unsupervised ML approach, hierarchical clustering, was utilized for hierarchical cluster analysis of IAV sequences. According to Equation 4, IAVs were clustered into various hierarchical groups based on the Euclidean distance in nucleotide compositional values.


(3) $$\begin{array}{l} \mathrm{minimize}\;\|A-XY{\|}_{F}^{2}=\overset{m}{\underset{i=1}{\Sigma }}\left\{\overset{n}{\underset{j=1}{\Sigma }}{\left(A_{ij}-x_{i}y_{j}\right)}^{2}\;\right\},\\ s.t.X\in R^{m\times k},Y\in R^{k\times n},k<m\;\mathrm{or}\;n \end{array}$$

(4) $$\begin{array}{l} \|a-b{\|}_{2}=\sqrt{\underset{i}{\Sigma }{\left(a_{i}-\mathrm{bi}\right)}^{2}},a,b=\mathrm{avian},\\ \mathrm{human}\;\mathrm{nt}\;\mathrm{features},i=1,2,\ldots ,9 \end{array}$$

Multiple Layer Perception Classifier (mlp) and Random Forest Classifier (rfc) were utilized, respectively, for supervised machine learning analysis, with “sklearn.neural_network.MLPClassifier” and “sklearn.ensemble.RandomForestClassifier.” Data were split into five training/test sets with sklearn.model_selection (n_splits = 5, random_state = 1, shuffle = True) for ML analysis. Scipy package (cluster.hierarchy, version 0.19.0, https://www.scipy.org) was utilized to build a hierarchical clustering of IAV sequences based on the Euclidean distance between/among sequences.

### 2.5 Adaptation risk assessment of simulated IAVs with pd09H1N1 EPplus and non-H1N1 RNPplus

A total of 221,184 simulated IAVs with pd09H1N1 EPplus and non-H1N1 RNPplus were analyzed for their adaptation to humans. First, five trained mlp predictors and five rfc predictors with an area under the receiver operating characteristic (ROC) curve AUC value of more than 0.98 and adaptation probability of more than 0.5 as thresholds. An adaptation score for simulated IAVs was set as the median value of the prediction results of the trained five mlp predictors and rfc predictors. The adaptation ratios (adapted/total) for simulated IAVs based on varied serotypes, country/area, and years were analyzed.

## 3 Results

### 3.1 Prediction pipeline and species-specific genomic nucleotide composition in IAVs

**Figure 1:**
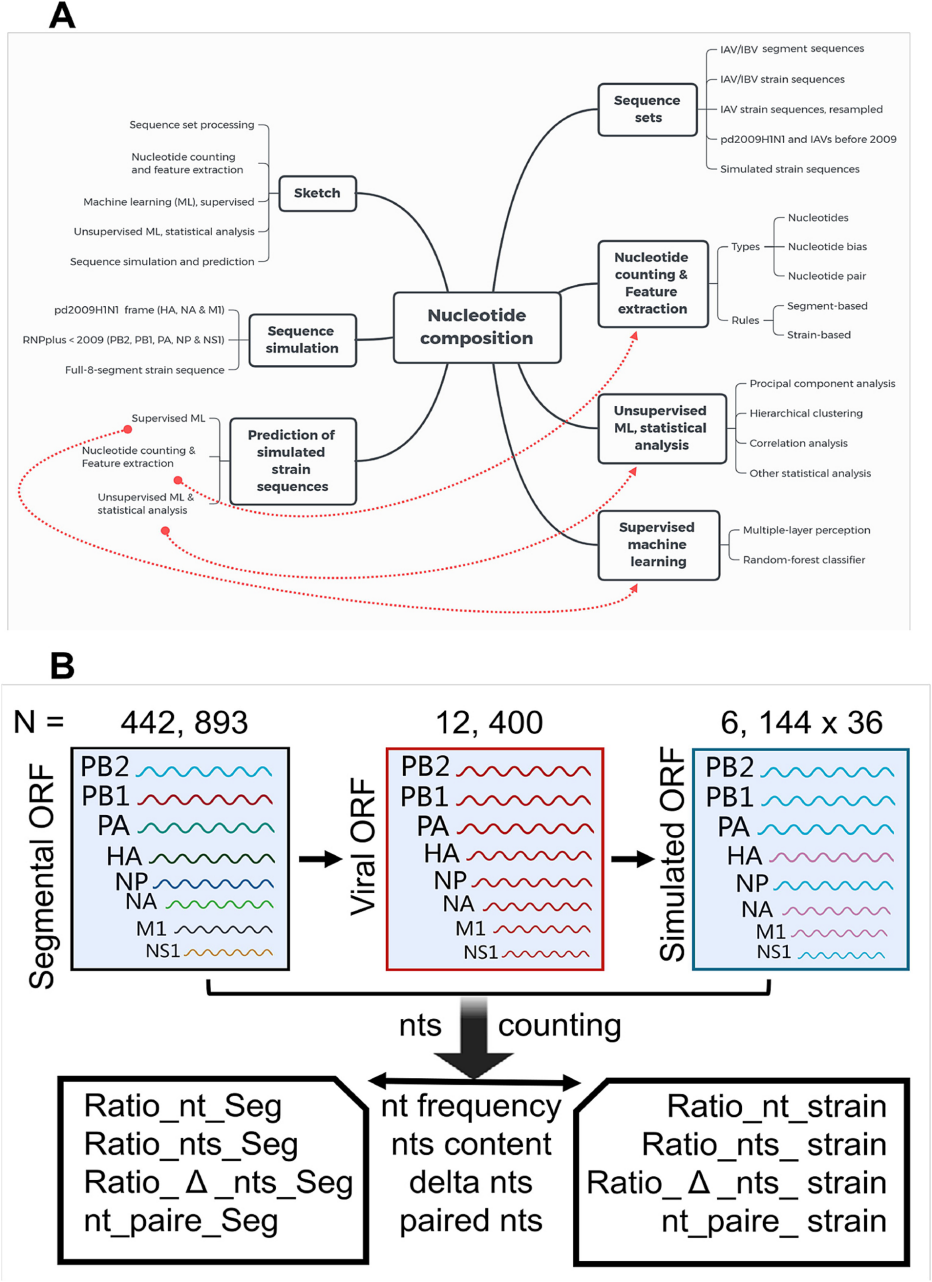
The workflow of data processing, machine learning analysis, and sequence simulation. **(A)** Workflow of data processing, machine learning analysis, and sequence simulation. Influenza A and B virus sequences were assembled on the turn (*PB2*, *PB1*, *PA*, *HA*, *NP*, *NA*, *M1*, and *NS1* successively) of segment number into a whole viral genomic sequence. Nucleotide composition was counted and analyzed with unsupervised and supervised approaches. The reassortment between the pd09H1N1 IAVs and the IAVs before 2009 were simulated, and human adaption of simulated IAVs was predicted with the aforementioned supervised machine-learning approach. **(B)** Sketch of the decomposition and simulation of IAV segmental and viral sequences. A total of 442,893 segmental (*PB2*, *PB1*, *PA*, *HA*, *NP*, *NA*, *M1*, and *NS1*) open reading frame (ORF) sequences were utilized to assemble, with all the eight segmental sequences from the same stain, the viral strain ORF sequences (*N* = 12, 400). The sequence simulation was performed to reconstruct the strain ORF sequences, with *HA*, *NA*, and *M1* from 36 pd09H1N1 IAV strains, and with *PB2*, *PB1*, *PA*, *NP*, and *NS1* from the 6,144 IAV strains before 2009. Frequency of four types of nucleotides (Ratio_nt_Seg, nt = t, c, a or g, Seg = *PB2*, *PB1*, *PA*, *HA*, *NP*, *NA*, *M1*, or *NS1*), The cg at content (Ratio_nts_Seg, nts = cg or at), the nucleotide bias (Ratio_Δ_cg_Seg/_strain and Ratio_Δ_at_Seg/_strain, relative number difference between a and t, between c and g), and paired nts (theoretically paired at and paired cg, nt_pair_Seg, nt_pair_stain) were counted as relative levels, dependent on segment- or strain.

Segment sequences from the same IAV stain were downloaded and were assembled on the turn of segment number (first to eighth: *PB2*, *PB1*, *PA*, *HA*, *NP*, *NA*, *M1*, and *NS1*) into a whole viral genomic coding sequence.12,400 IAV strains with full eight-segment coding sequences were assembled (Supplementary Figure S1). A stochastic resampling was performed to reduce the distribution bias on the USA of the Country_area label and on the post-2009 of the Year label (Supplementary Figure S2). For the total of 9,525 strains, 2,372 samples from the USA and 2,230 samples from China accounted for half of the total samples, 4,500 strains were from mammalian hosts (2,978 from humans and 1,522 from swine), and the remainder 5, 025 were from avian hosts (Supplementary Figures S2A,B). The strain samples for each category of subtype and year labels were also presented (Supplementary Figures S2C,D). As shown by the pipeline diagram (Figure 1), machine learning models were built based on the nucleotide composition of human and avian IAV sequences to discriminate the IAV human adaption. Segment sequences with different subtypes and host labels were utilized to simulate IAV reassortment and the learning models were used to predict the human adaptation probability of such simulated reassortment.

**Figure 2:**
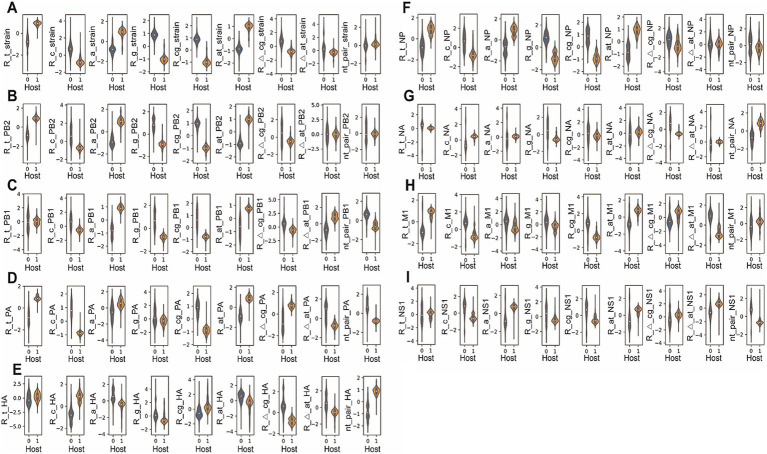
Violin plot of the nucleotide composition factors for avian and mammalian influenza A viruses. The frequency of nucleotide t, c, a or g (R_t, R_c, R_a or R_g), the frequency of gc or at content (R_at_ or R_cg), the relative levels of nucleotide bias (R_Δ_at or R_Δ_cg) and of nucleotide pair (nt_pair) were counted strain-dependently or segment-dependently (*PB2*, *PB1*, *PA*, *HA*, *NP*, *NA*, *M1*, or *NS1*), respectively **(A–I)**; relative frequency value was plotted with Violin plot (seaborn model, Python); data were standardized as (value – value mean)/value SD. 0: Avian IAVs, 1: Mammalian IAVs. A *p*-value for each factor was indicated independently.

The virus nucleotide composition was analyzed based on a segment or based on a strain. The average level and the distribution of each nucleotide composition item (except strain nt pair, the last subplot in Figure 2A) were plotted, respectively, for virus strain or virus segment (Figures 2A–I), showing a statistical difference between mammalian (human and swine) and avian hosts (*p* < 0.001 except for strain nt-pair, Supplementary Table S1). The higher Ratio_t, low Ratio_c, higher Ratio_a, lower Ratio_g, lower Ratio_cg, and higher Ratio_at were unanimously observed for strain sequence, *PB2*, *PB1*, *PA*, *NP*, *M1*, and *NS1* in the mammalian IAVs, compared to avian IAVs (*p* < 0.001 respectively, Figures 2A–D,F,H,I). To associate the nucleotide composition with host species, we also analyzed such nucleotide composition differences between IAVs and IBVs, the latter of which only infect human hosts (Long et al., 2019). Interestingly, the nucleotide composition difference between IBV and IAV was the same as the difference between mammalian IAVs and avian IAVs (Supplementary Figures S3A–H; Supplementary Table S2). Such bias was also found for these segments (except Ratio_c_*NP*, Ratio_t_*M1*, and Ratio_t_*NS1*) in IBVs, compared to IAVs (*p* < 0.001 respectively, Supplementary Figures S3A–D,F,G). Besides, Hierarchical clustering was performed to evaluate the separability of nucleotide composition between avian and human IAVs. It was indicated that the random-sampled human and avian segment sequences were automatically separated into human and avian groups, except for some environmental H7N9 IAVs in a human group, and some human-infected avian IAVs and some 1968’s H3N2 viruses in the avian group (Supplementary Figures S4A–D for *PB2*, *PB1*, *PA*, and *HA*; Supplementary Figures S5A–D for *NP*, *NA*, *M1*, and *NS1*). Therefore, the nucleotide composition is host specific.

### 3.2 Intersegment nucleotide composition correlation of IAVs

To visualize the correlation among nucleotide composition features for IAV strains and the separability of each feature between avian and human samples, every pair of the nine features was plotted in a two-dimensional space. When Ratio_t_*PB2* was taken as an x-axis label, each of the other eight features was separable for *PB2* between avian and human samples (Supplementary Figure S6A), and some features presented a linear distribution, negatively (R_c_*PB2*, R_g_*PB2*, R_cg_*PB2*, and R_Δ_at_*PB2*) or positively (R_a_*PB2* and R_at_*PB2*). The Spearman rank correlation analysis indicated a significant negative correlation between Ratio_t_*PB2* and each of the four features (R_c_*PB2*, R_g_*PB2*, R_cg_*PB2* and R_Δ_at_*PB2*) (*R*^2^ < −0.3 respectively, firstly column in Supplementary Figure S6B) and a significant positive correlation between Ratio_t_*PB2* and each of the two features (R_a_*PB2* and R_at_*PB2*) (*R*^2^ > 0.3 respectively, firstly column in Supplementary Figure S6B). The avian/human separability and the negative or positive correlation were also observed for other features (other columns in Supplementary Figures S6C,D) or other segments (Supplementary Figures S7–S9).

**Figure 3:**
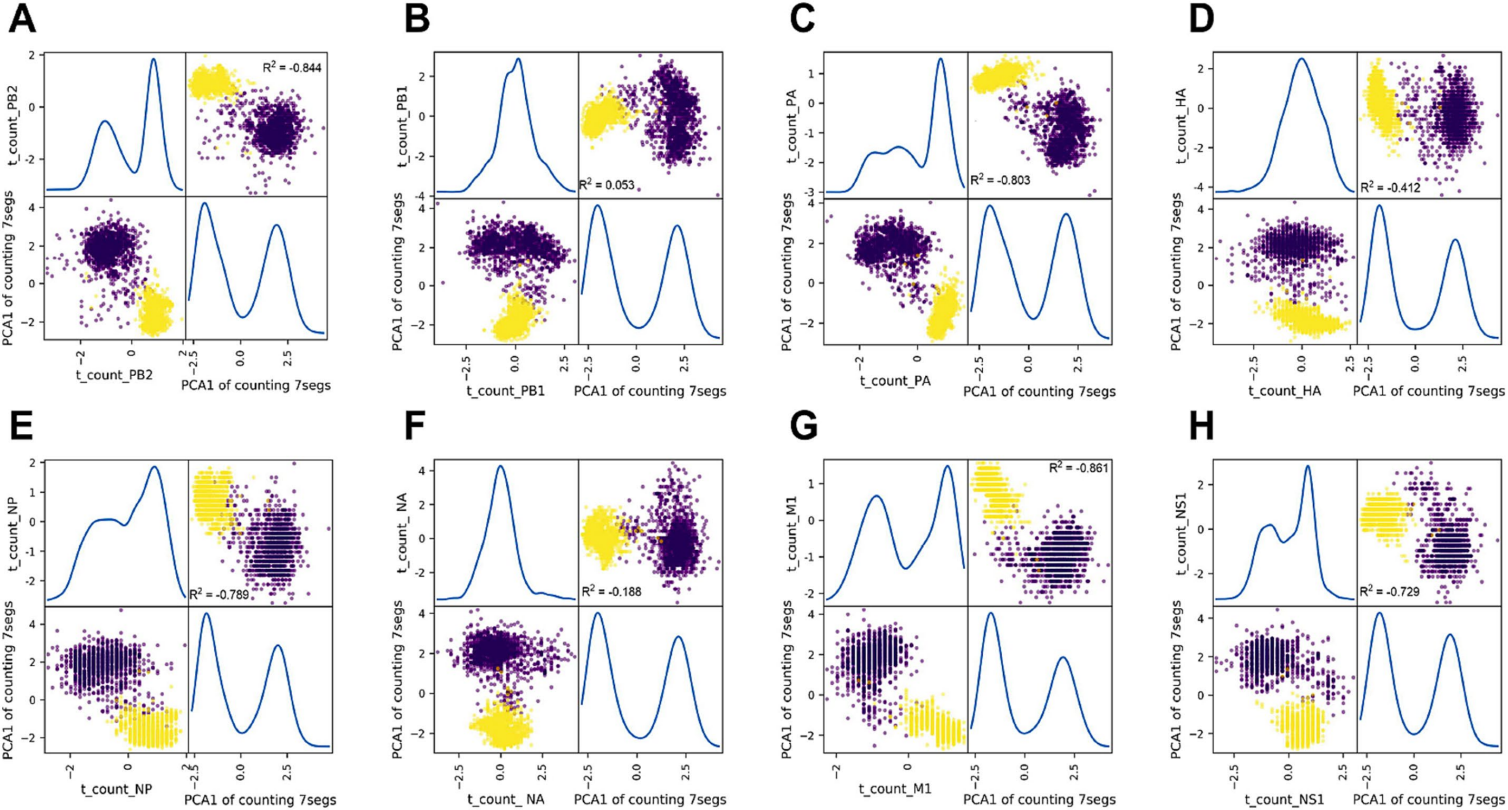
Principal component analysis (PCA) of thymine composition between each segment and the other seven segments for IAVs. Thymine composition (Ratio_t) for every seven segments (**A–H** for *PB2* and the other seven segments) was converted into one principal component (PCA model from sklearn.decomposition.PCA), along with Ratio_t_*PB2*, were scattered with scatter_matrix (pandas.plotting, Python). The correlation of the Ratio_t between each segment and the PCA1 of the other seven segments or the correlation of the two PCA1 for both groups of segments were analyzed with the Pearson correlation model of Pandas (pandas.DataFrame.corr (method = ‘Pearson’)) and were indicated as *R*^2^, respectively. Data of nucleotide ratio were standardized as (value – value mean)/value SD. 0.3 and −0.3 were set as the threshold of *R*^2^, respectively, for positive and negative correlation.

The correlation between segments for each nucleotide composition feature was also analyzed using principal component analysis (PCA). Ratio_c_*PB2* served as a label for every strain sample, and the Ratio_c matrix for the remaining seven segments was reduced into one principal component (PCA1_7segs) by PCA. The paired plotting of Ratio_t_*PB2* and PCA1_7segs in Figure 3A demonstrated a significant negative correlation (*R*^2^ = −0.844). A negative or positive correlation was also observed (Figures 3B–H) between the Ratio_c of each PCA1_7segs of the rest segments (*R*^2^ < −0.3 or *R*^2^ > 0.3), except *PB1* and *NA* (*R*^2^ = 0.053 for *PB1* and *R*^2^ = −0.188 for *NA*). The intersegment correlation was also significantly different for c_count, a_count, or g_count between each of the eight segments and the PCA1_7segs value, except for the g_count of *HA* and *NS1* (*R*^2^ < −0.3 or *R*^2^ > 0.3, Supplementary Figures S10–S12).

**Figure 4:**
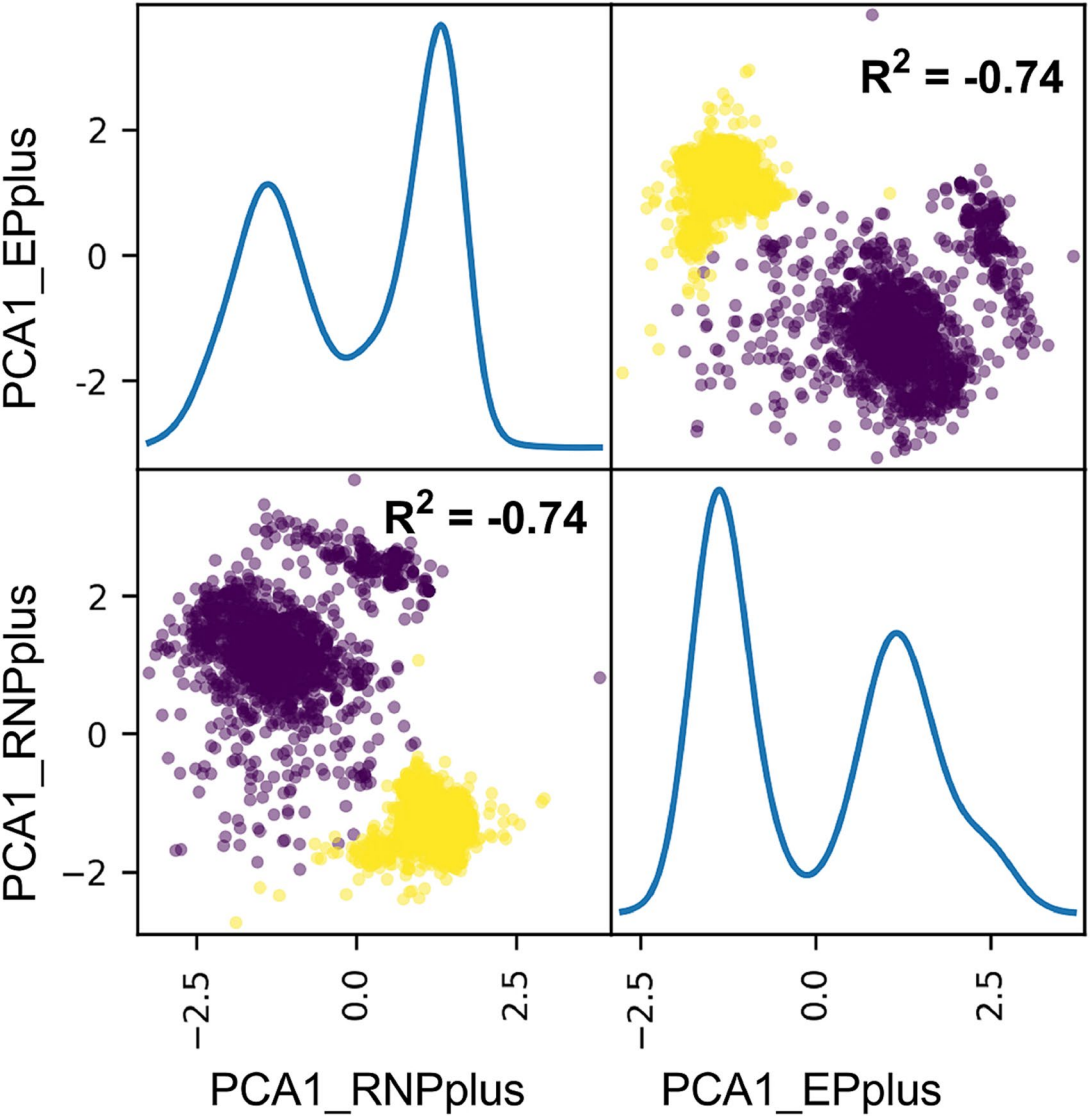
Principal component analysis (PCA) of nucleotide cytosine composition between RNPplus and EPplus for IAVs. The nt_pair value for the segments of *PB2*, *PB1*, *PA*, *NP*, and *NS1* (RNPplus) and for the segments of *HA*, *NA*, and *M1* (EPplus) were, respectively, converted into one principal component and then were scattered. The distribution of the two values for avian and human sequences was scattered in brown and yellow, respectively.

Interestingly, there was a similarity in the distribution of the correlation coefficient matrix for segments *PB2*, *PB1*, *PA*, *NP*, and *NS1* (Supplementary Figures S6B,D, 7B, 8B, 9D), based on the polarity and the degree of such correlation. All nucleotide composition features for *PB2*, *PB1*, *PA*, *NP*, and *NS1* (ribonucleoprotein plus [RNPplus]) were reduced into one PCA component, and these features for *HA*, *NA*, and *M1* (Envelope Protein, EPplus) were reduced into another PCA component. There was a strong negative correlation between the two components and also an indication of separability between avian and human samples (*R*^2^ = −0.74), as shown in Figure 4. These results reveal the intersegment nucleotide composition correlation of IAVs.

### 3.3 Human adaption prediction of IAVs based on nucleotide composition

**Figure 5:**
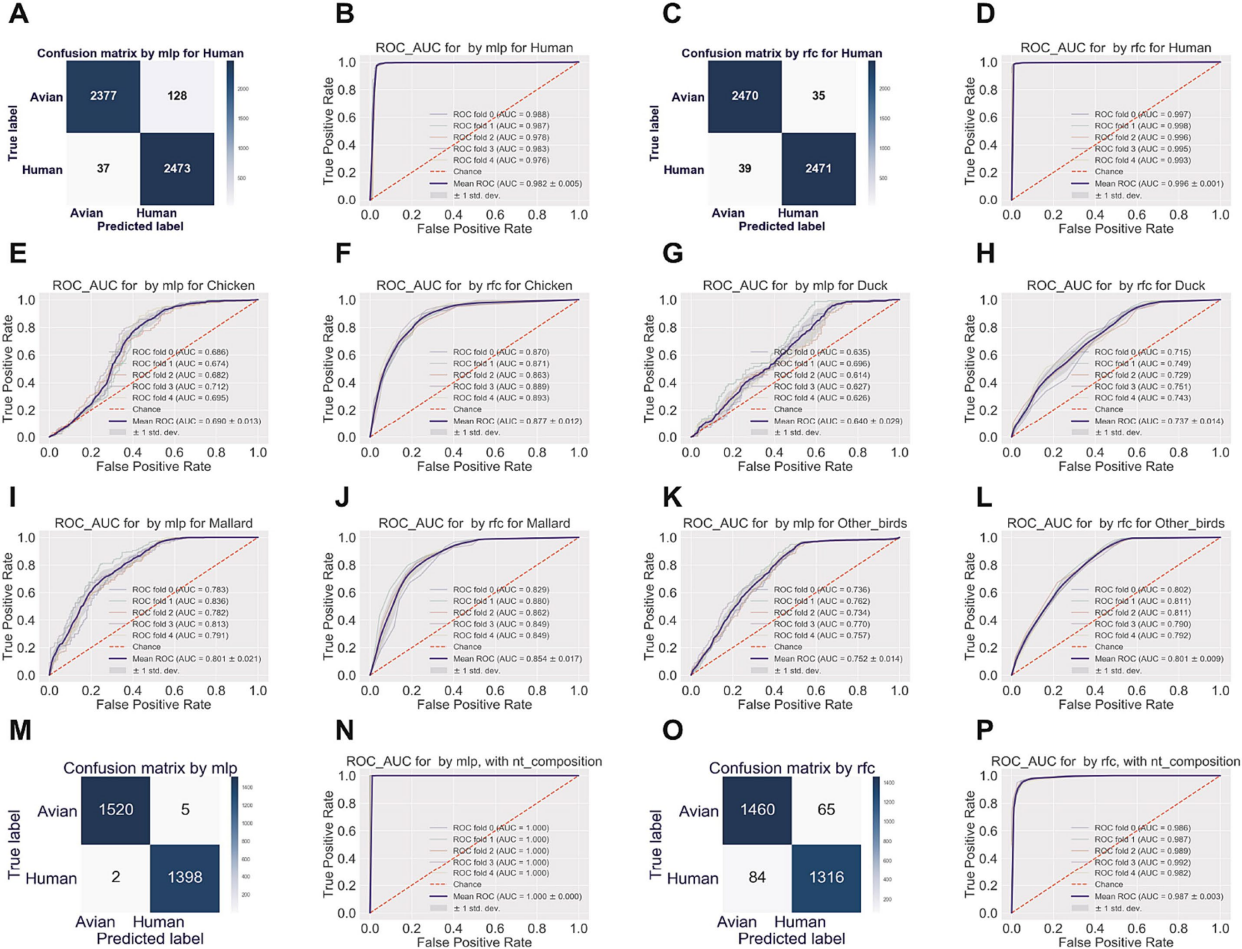
Human adaptation prediction by machine-learning approaches, with nucleotide composition factors. The prediction and the probability of virus adaption to humans were evaluated by supervised machine learning approaches of random forest classifier (rfc) **(A,B)** and multiple-layer preceptor (mlp) **(C,D)**. The receiver operating characteristic (ROC) and area under the ROC curve (AUC) **(B,D)** and the confusion matrix **(A,C)** of human adaption prediction were indicated, respectively. Training data were randomly split into five folds; 1x standard deviation (±1 SD) was adopted for ROC and AUC. ROC_AUC of the mlp or the rfc to discriminate the chicken- (**E,F**, respectively for mlp and rfc), duck- **(G,H)**, mallard- **(I,J)**, or other birds-originated **(K,L)** IAVs from the IAVs from the rest three avian types of hosts. Confusion matrix and ROC_AUC were plotted of the mlp **(M,N)** or the rfc model (**O** or **P**) for simulated reassortant H1N1 viruses, with segments from the pd09H1N1 virus and with segments from other subtypes of IAVs.

Multiple-layer perceptron (mlp) and random forest classifier (rfc) were utilized as supervised machine-learning approaches to predict the human-adaptive IAVs (H3N2 and H1N1) from avian IAVs (H5N1, H9N2, and H7N9), based on nucleotide composition features. It was shown that the true negative rate (true prediction of avian IAVs) and the true positive rate (true prediction of human IAVs) were 94.89% (2,377/2,505) and 98.53% (2,473/2,510), respectively, for mlp model (Figure 5A). The mean AUC of 5-fold tests was 0.982 ± 0.005 (Figure 5B). The rfc model performed as well as mlp model, with true negative/positive rates of 98.60% (2,470/2,505) and 98.45% (2,471/2,510), respectively (Figure 5C), and with the mean AUC of 0.996 ± 0.001 (Figure 5D).

ML models performed well in discriminating human IAV sets from a mixed IAV set from chicken, duck, mallard, and other avian hosts. To test whether such high performance was associated with the uniformity of the human dataset and the mixing property of the avian dataset, we performed mlp and rfc analyses to discriminate between the set of IAVs from chicken, duck, mallard, or other birds from the set of IAVs from the rest three types of avian hosts and from humans. As indicated in Figures 5E–L, the mean AUC only reached 0.690 ± 0.013, 0.640 ± 0.029, 0.801 ± 0.021, and 0.752 ± 0.014 by mlp model for each of the four avian hosts, the mean AUC reached to a little higher level, but not yet over 0.900 (0.877 ± 0.012, 0.737 ± 0.014, 0.854 ± 0.017 and 0.801 ± 0.009, respectively) by rfc model for each of the four avian hosts. Therefore, viral nucleotide composition accurately predicts the human adaption of IAVs using machine learning models. Additionally, we utilized the two models, with only human H3N2 IAVs as the human set in training data, to predict human H1N1 IAV. Both mlp and rfc models performed well for the H1N1 IAV prediction (Supplementary Figures S13A,B).

### 3.4 Human adaption prediction of reassortment pd09H1N1 IAVs based on the intersegment nucleotide composition correlation

The 2009 H1N1 influenza pandemic, the most recent influenza pandemic, was caused by a reassortment virus that contained segments of avian-, swine- and human-originated (Smith et al., 2009). To predict the reassortment of the pd09H1N1 virus with other IAVs, we built the mlp and rfc models with the human IAV set of one of the two major human IAV subtypes (H3N2 and H1N1), H3N2, and with the avian IAV set of dominant avian subtypes of H5N1, H9N2, and H7N9. As shown in Figures 5M–P, both models performed well in predicting the human adaption of the above-mentioned human IAVs. With the rcf model, we predict the human adaption of simulated reassortant H1N1 viruses, with segments from the pd09H1N1 virus and with segments from other subtypes of IAVs.

The reassortment between the pd09H1N1 virus and other subtypes of IAVs was simulated based on the uniform difference for RNPplus between avian and mammalian IAVs (Figure 2) and on the negative nucleotide composition correlation between RNPplus and EPplus (Figure 4). Thirty-six human-originated H1N1 strains isolated in 2009 in the USA were taken as pd09H1N1 viruses; 6,144 avian IAVs of the subtypes, other than H1N1 were taken as no-pd09H1N1 IAVs. A total of 221,184 reassortant H1N1 viruses with *HA*, *NA*, and *M1* from pd09H1N1 viruses, and with the other five segments from no-pd09H1N1 IAVs were produced. Sample distribution on the label of Country_area, Host, Subtype, and Year was indicated, respectively (Supplementary Figure S14).

To interpret the significance of genomic NC to the IAV adaptation classification, dimension reduction by PCA of the optimized NC features was performed and plotted with a pairplot with host labeled. A distinct separation of PCA1 value between human and avian hosts was indicated (Supplementary Figure S15A). In contrast, the PCA1 value of the 3,721-dimensioned codon-pair was not markedly separated between human and avian simulated IAVs; only with PCA2 value was separated (Supplementary Figure S15B). Both types of results implied a higher significant difference in NC features between human and avian simulated IAVs than in codon-pair.

**Figure 6:**
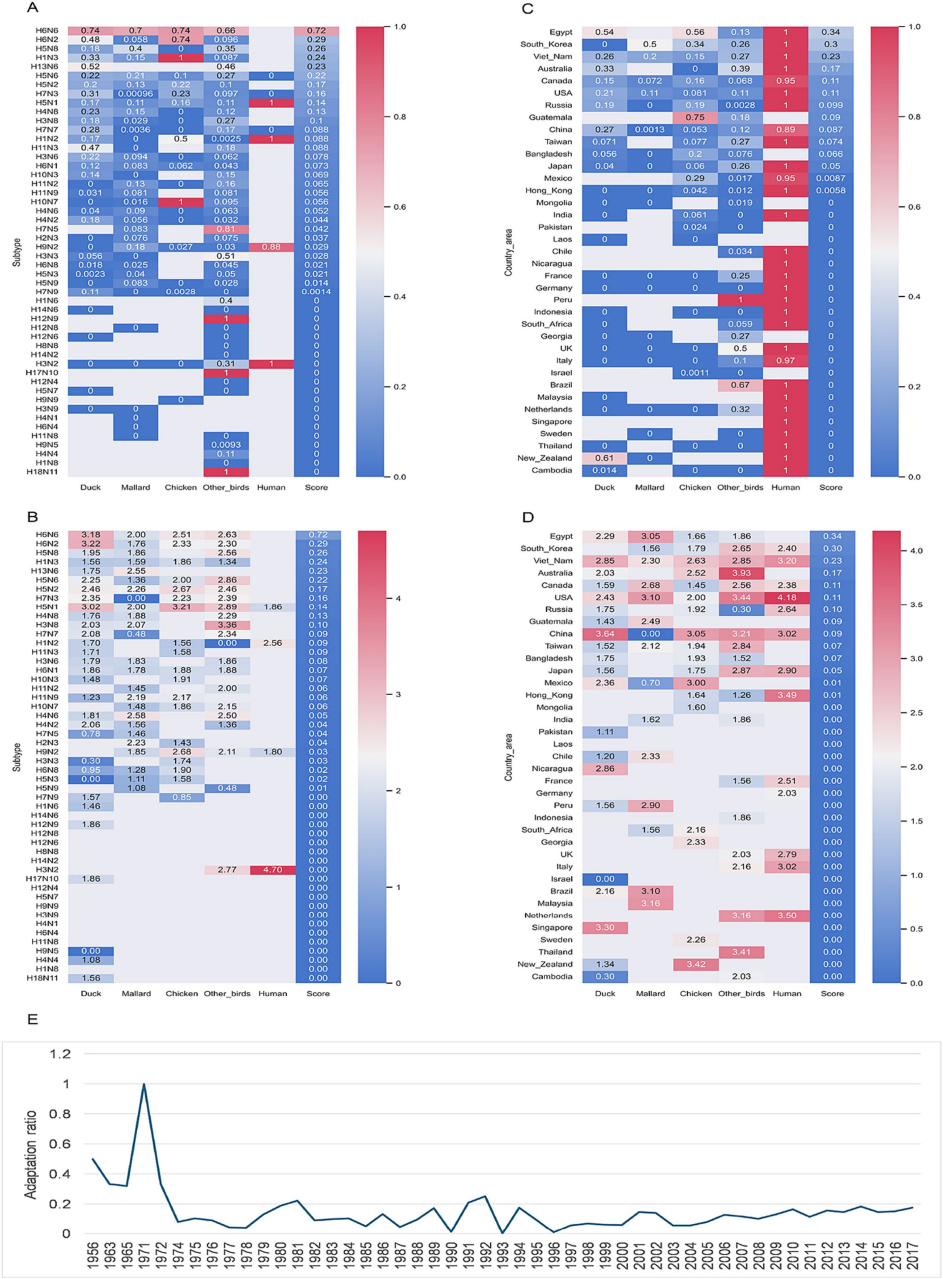
Adaptation prediction of the simulated IAV reassortants with pd09H1N1 EPplus and non-H1N1 RNPplus. Heatmap of adaptation ratio (adapted/total) **(A)** and adapted numbers (presenting as ln(adapted number)) **(B)** for the simulated IAVs with RNPplus from the IAVs from top 50 serotypes, top 37 (more than 500 IAV samples) country/areas **(C,D)** or top 50 years **(E)**.

Human adaption of these simulated IAVs was predicted by both mlp and rfc models, with the nucleotide composition features. Adaptation risk for each simulated reassortant was evaluated by a risk score, which was calculated based on the adaptation prediction results of five mlp predictors and five rfc predictors. Both adaptation ratio or adapted number indicated a high adaptive reassortment with pd09H1N1 EPplus of the RNPplus from the IAVs of such serotypes as H6N6, H6N2, H5N8 and others [adaptation ratio and adapted number, respectively (Figures 6A,B; Table 1)]. Both adaptation indexes indicated a high adaptation risk in Egypt, South Korea, Vietnam, Australia, and Canada (adaptation ratio and adapted number, respectively, in Figures 6C,D; Supplementary Table S3), with other top countries/areas also listed. The temporal adaptation ratio of these simulated reassortants (Figure 6E) showed a steep rise before 1971 and a followed outstanding peak in 1971. A waving adaptation ratio of IAV RNPplus has been lasting since the 1970s to now. It’s worth mentioning that a slow but sustained adaptation rise has been observed since 2004.

**Table 1:** Adaptation ratio (adapted/total) of simulated reassortants between pd09H1N1 EPplus and the IAV of varied serotypes.

Subtype^a^	Duck	Mallard	Chicken	Other_birds	Human	Score^b^
H6N6	0.740	0.701	0.741	0.660		0.721
H6N2	0.477	0.058	0.736	0.096		0.286
H5N8	0.177	0.400	0.000	0.347		0.262
H1N3	0.333	0.155	1.000	0.087		0.244
H13N6	0.519			0.464		0.232
H5N6	0.223	0.213	0.103	0.267	0.000	0.218
H5N2	0.204	0.134	0.218	0.104		0.169
H7N3	0.313	0.001	0.226	0.097	0.000	0.162
H5N1	0.167	0.106	0.163	0.108	1.000	0.135
H4N8	0.226	0.151	0.000	0.119		0.135
H3N8	0.176	0.029	0.000	0.268		0.102
H7N7	0.280	0.004	0.000	0.173	0.000	0.088
H1N2	0.174	0.000	0.500	0.003	1.000	0.088
H11N3	0.472	0.000		0.176		0.088
H3N6	0.215	0.094	0.000	0.063		0.078
H6N1	0.125	0.083	0.062	0.043		0.073
H10N3	0.139	0.000		0.150		0.069
H11N2	0.000	0.130	0.000	0.163		0.065
H11N9	0.031	0.081		0.081		0.056
H10N7	0.000	0.016	1.000	0.095		0.056

a Top-20 subtypes with high human probability were listed.

b A score of adaptation risk was set as a median adaptation ratio of simulated IAVs with RNPplus from various hosts from the same serotype.

## 4 Discussion

Lots of viral protein determinants have been identified in host tropism (Eng et al., 2016), trans-species infection (Qiang et al., 2018), and virulence (Li et al., 2011; Oxford and Gill, 2018; Tscherne and Garcia-Sastre, 2011). Recent reports indicate the functional importance of viral nucleotide composition. Synonymous viral nucleotides or dinucleotides regulate the virus’s response to the host’s innate immune system (Takata et al., 2017), affect virus virulence (Atkinson et al., 2014; Tulloch et al., 2014), and influence virus replication (Witteveldt et al., 2016). The host dependence of the nucleotide compositions of influenza viruses has also been implied (Bahir et al., 2009; Iwasaki et al., 2013; Su et al., 2009). However, the reliance of host species on nucleotide composition was not supported by other studies (Di Giallonardo et al., 2017). In this study, we calculated the nucleotide composition based on each segment and also based on the entire genome by counting the frequency of each mononucleotide, the content of gc and at, the surplus of paired t/a and of paired c/g, and the paired nucleotides (t/a and c/g). We found a uniform difference between avian and mammalian IAVs, between the only-human-infected (Long et al., 2019) IBVs and the IAVs infect both birds and mammals. The higher Ratio_t, low Ratio_c, higher Ratio_a, lower Ratio_g, lower Ratio_cg, and higher Ratio_at were unanimously observed for the entire genomic sequence, *PB2*, *PB1*, *PA*, *NP*, *M1*, and *NS1* in the mammalian IAVs and IBVs, compared to avian IAVs, or all IAVs. The unsupervised machine-learning approach of hierarchical clustering and the supervised machine-learning approaches of rfc and mlp unanimously confirmed the separability based on nucleotide composition between avian and human IAVs. Therefore, the nucleotide composition of IAVs is host specific.

There has not been a widely accepted definition of human adaptation for IAVs, and here we defined it as the capability to infect humans easily and to transmit among the population efficiently. The nucleotide composition of IBVs may represent a human-adaptive feature as IBVs are specifically adapted to humans and spread exclusively among humans. More importantly, the nucleotide composition features of IBVs were uniform for six of eight genomic segments, except *HA* and *NA*; such uniform features were also found from these segments for mammalian IAVs, compared to avian IAVs. *HA* and *NA* are primary targets for an adaptive immune response to influenza infection (Andrews and McDermott, 2018; Bahadoran et al., 2016). There is a higher mutation rate in *HA* and *NA* under the host immune pressure compared to the other six segments (Ridenour et al., 2015; Xu et al., 1996). We speculated that the nucleotide composition of *HA* and *NA* was more influenced by host immune pressure, than the other six segments. Therefore, currently, human-adaptive IAVs are limited to H3N2 and H1N1 viruses, either of which continuously cause endemics or even pandemics in humans (Ren et al., 2016). To avoid possible overfitting for the subsequent prediction of simulated reassortant pd09H1N1 IAVs, human H1N1 viruses were not included in the training set; thus, the performance of our models in predicting the human adaption of the H1N1 viruses since 2009 was comparable to that of H3N2 viruses.

The mechanism underlying the high intersegment reassortment of IAVs is not well understood. It appears that a reassortment does not occur randomly, but rather tends to involve specific segments (Marshall et al., 2013), according to observation and experimental results (Arai et al., 2019; Chen et al., 2008; Kimble et al., 2011; Octaviani et al., 2010). The compatibility or balance of viral proteins (Li et al., 2008; Naffakh et al., 2000; Wagner et al., 2002) is crucial for IAV reassortment. The incompatibility of RNA packaging signals in the segmental untranslated region (UTR) and parts of coding sequences restricts IAV reassortment (Cobbin et al., 2014; Essere et al., 2013). Here, we investigated the intersegment correlation of the nucleotide composition of IAVs. Each nucleotide composition feature correlated with the other features within a segment for each of the eight segments, and each segment correlated to the other segments in nucleotide composition to various degrees of IAVs. Moreover, there was a similarity in the distribution of the correlation coefficient matrix for segments *PB2*, *PB1*, *PA*, *NP*, and *NS1*, based on the polarity and the degree of such correlation. RNPplus, the PCA component 1 for nucleotide composition features of segments *PB2*, *PB1*, *PA*, *NP*, and *NS1*, negatively and strongly correlated with EPplus, the PCA component 1 for nucleotide composition features of *HA*, *NA*, and *M1*. Our results imply that the intersegment correlation of nucleotide composition might be another constraint factor for IAV reassortment.

The host also poses constraints on IAV reassortment via multiple mechanisms, via multiple mechanisms, such as antivirus immune response, whether innate or adapted, and receptor binding efficiency. Various types of host proteins regulate the activity of the IAV polymerase complex and thus constrain IAV reassortment (Tripathi et al., 2015). Human-receptor-bindable H3N8 viruses were transmissible among ferrets, facilitating possible reassortment between it and other human IAVs (Sun et al., 2023). The dynamics of IAV replication in mammals allow diversification through reassortment of variants, shaping their evolution and onward transmission (Ganti et al., 2022). Immune escaping of IAVs benefiting from IAV reassortment facilitates the selection of IAV reassortants within the host (Vijaykrishna et al., 2015). Additionally, tissue specificity was also observed to pose constraints on IAV reassortment (Tripathi et al., 2015). Given the challenge of analyzing interactively the constraints from host and viruses, this study only focused on the significance of viral genomic features on IAV reassortment.

pd09H1N1 virus caused the latest worldwide influenza pandemic (Fineberg, 2014; Swerdlow et al., 2011). Here, we simulated the reassortment of pd09H1N1 viruses with RNPplus from human and avian IAVs. Interestingly, the reassortment viruses containing RNPplus from human H3N2 and the EPplus from pd09H1N1 were not adaptive to humans. However, some subtypes of IAVs, such as H6N2, H5N6, H6N6, H5N8, H16N3, H13N6, H13N8, H13N2, and H5N5, all of which previously spread mainly in birds, provide the human-adaptive RNPplus against the backdrop of pd09H1N1 EPplus. Notably, the reassortment pd09H1N1 viruses, with the RNPplus from H6N6, H13N8, and H13N2, were mostly highly risky, with an AUC of more than 0.9. Such a high human adaption score should arouse alertness against such high-risk reassortment. Our simulation was performed with the 36 whole genome-sequenced pd09H1N1 viruses in the USA and with all the other subtypes of IAVs available in the influenza research database (IRD) (Zhang et al., 2017). The simulated virus numbers varied from 324 for H7N6 viruses to 53,316 for H3N2 viruses; the variation in the virus number reduces the prediction comparability among varied subtypes.

## 5 Conclusion

In summary, there is a human adaption-specific genomic nucleotide composition with which machine-learning approaches discriminate human IAVs from avian IAVs, accurately. The nucleotide composition correlates with others among different IAV segments and constrains segment reassortment from different subtypes of IAVs, such as pd09H1N1 viruses with other subtypes of viruses. Machine learning analysis with viral nucleotide composition provides a novel strategy to predict or evaluate the human adaption of IAVs.

## Data Availability

The datasets presented in this study can be found in online repositories. The names of the repository/repositories and accession number(s) can be found in the article/Supplementary material. Codes for this study was available on GitHub (https://github.com/Jamalijama/IAVreassormentConstraint). All raw data and any information about the methodology and results of this work is available upon request from the lead contact (Jing Li, lj-pbs@163.com).
