# Supplementary material for: Machine learning methods for predicting human-adaptive influenza A virus reassortment based on intersegment constraint
Source: Front Microbiol. 2025 Mar 21;16:1546536. doi: 10.3389/fmicb.2025.1546536 (PMC11970406; doi:10.3389/fmicb.2025.1546536)

Suppl Figure 1

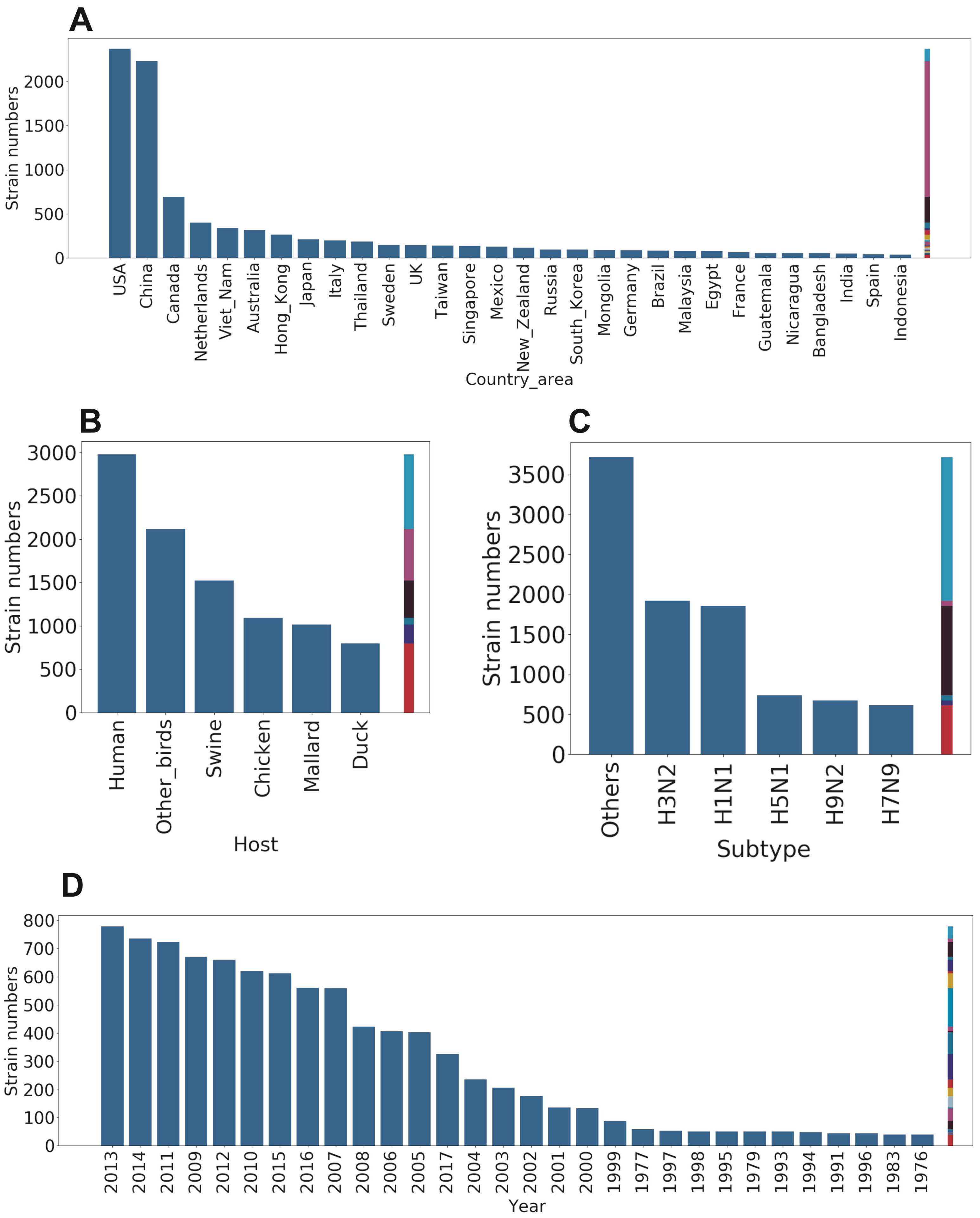

Suppl Figure 2

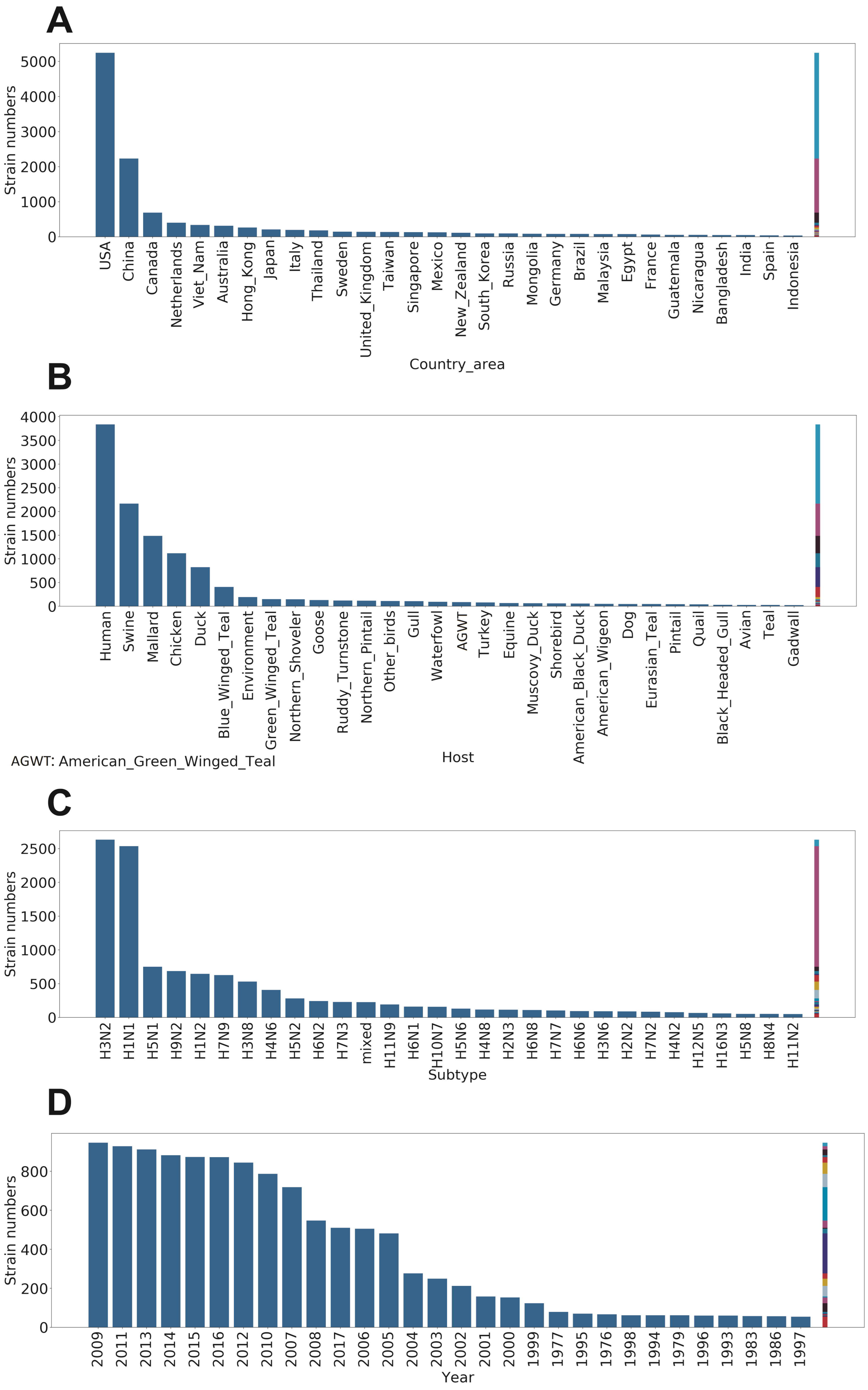

Suppl Figure 3

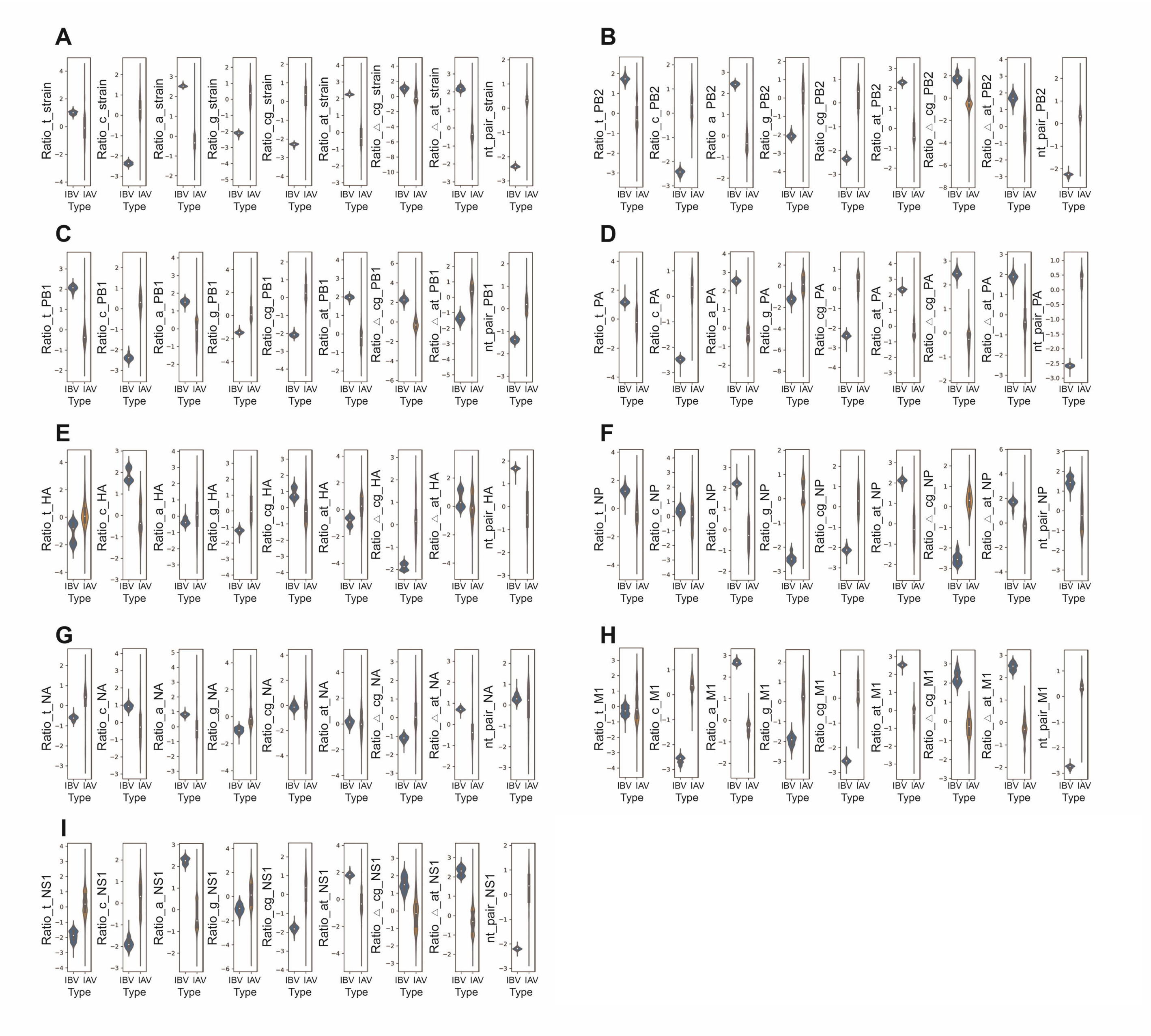

Suppl Figure 4

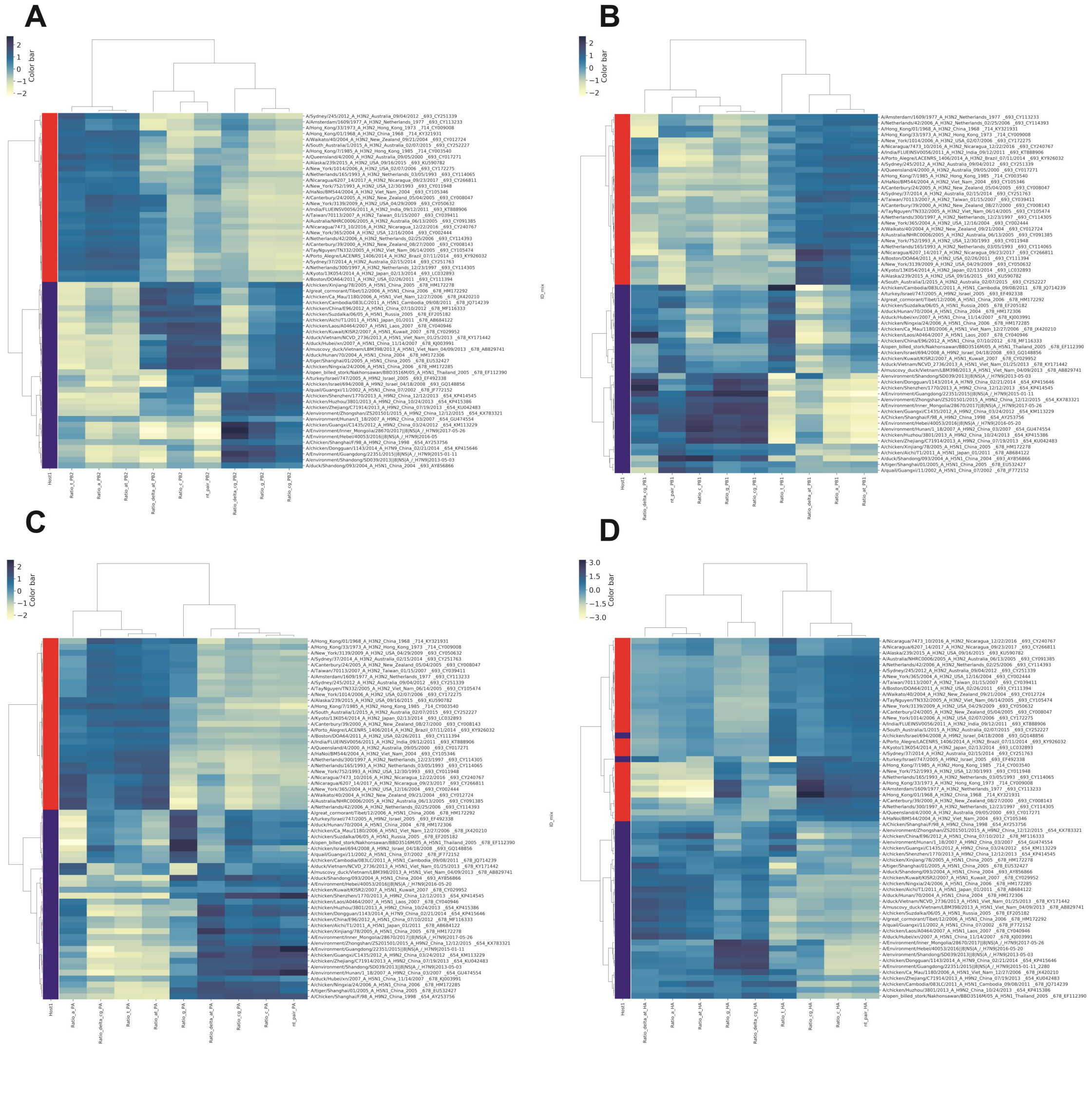

Suppl Figure 5

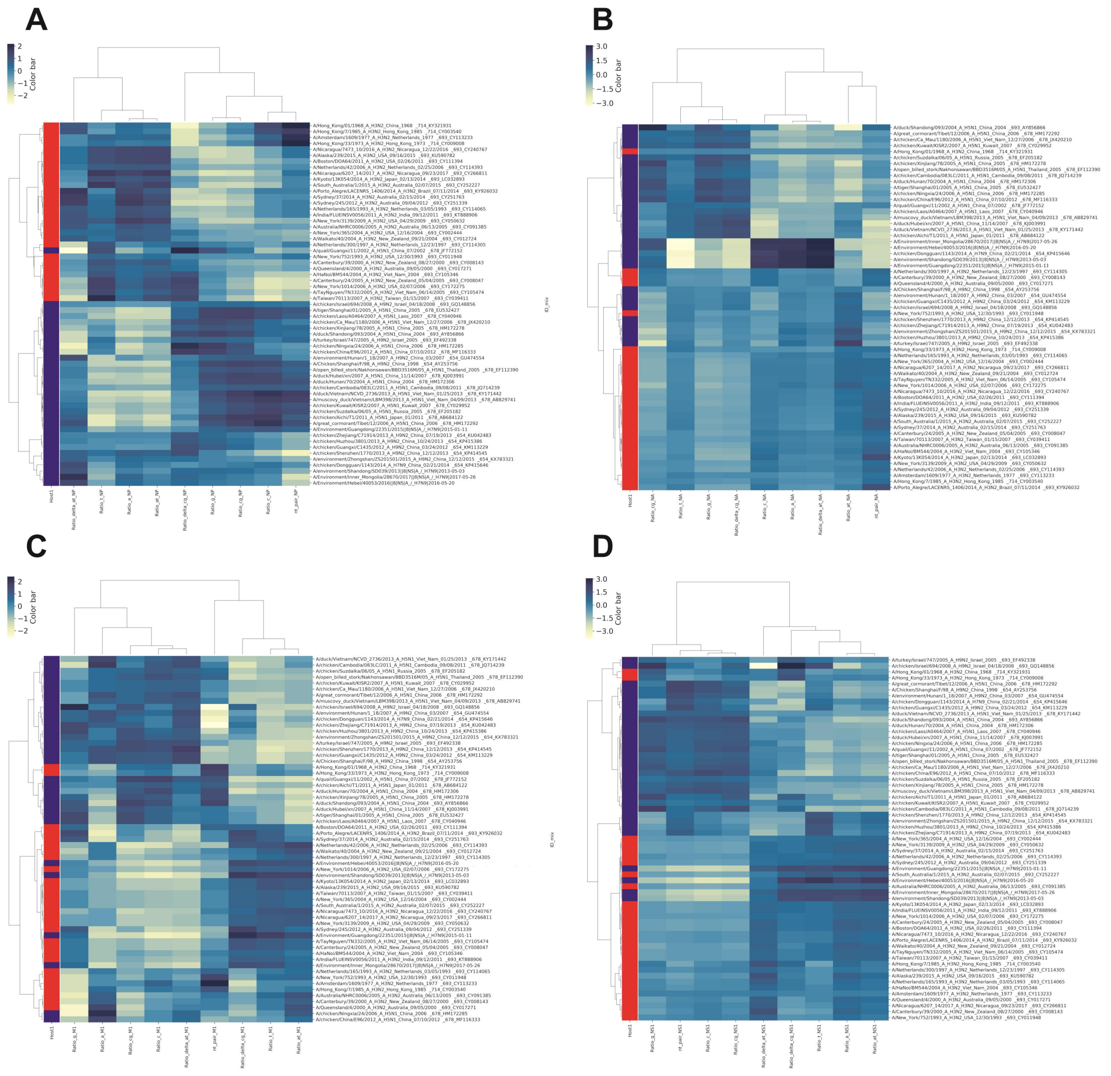

Suppl Figure 6

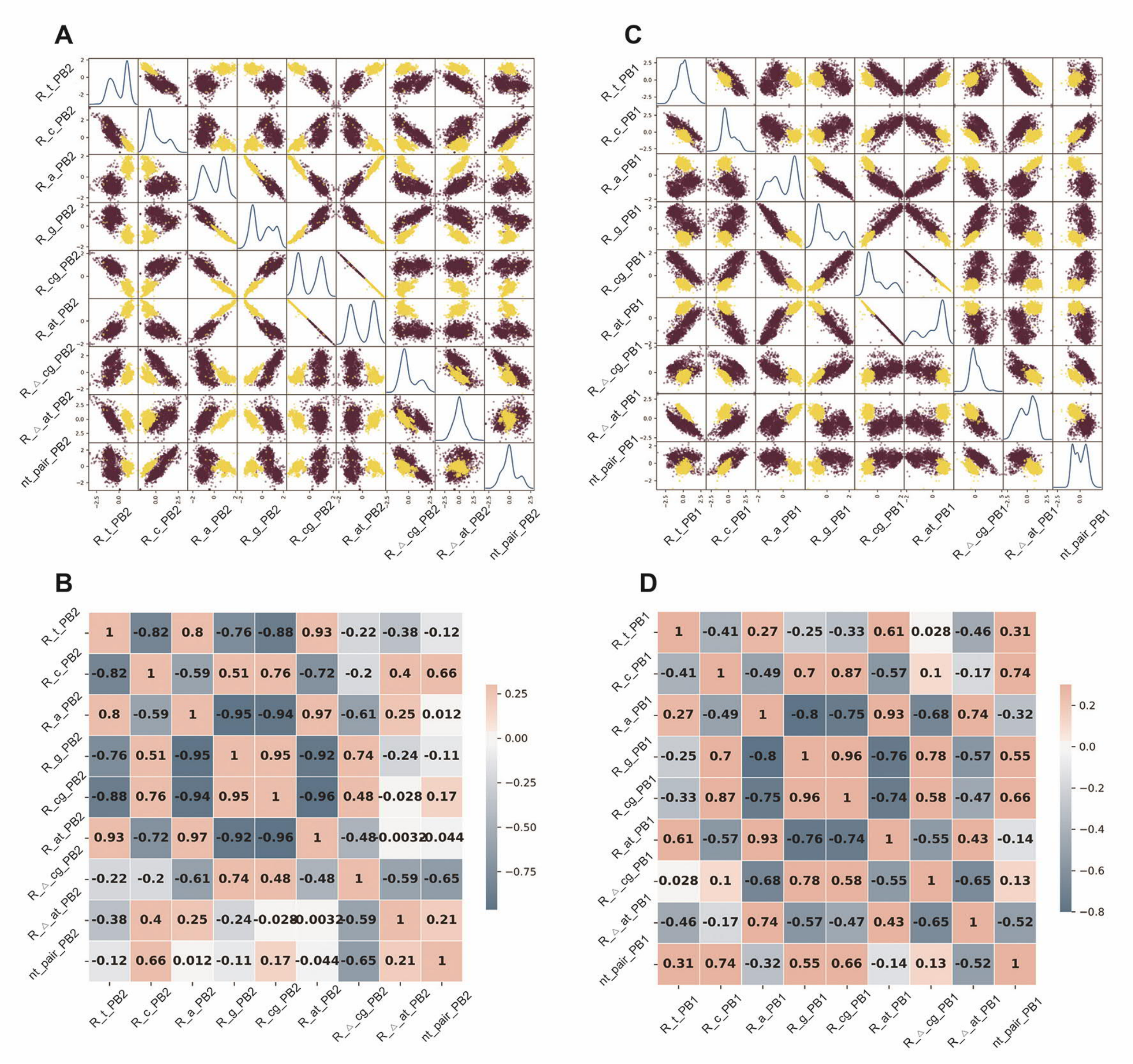

Suppl Figure 7

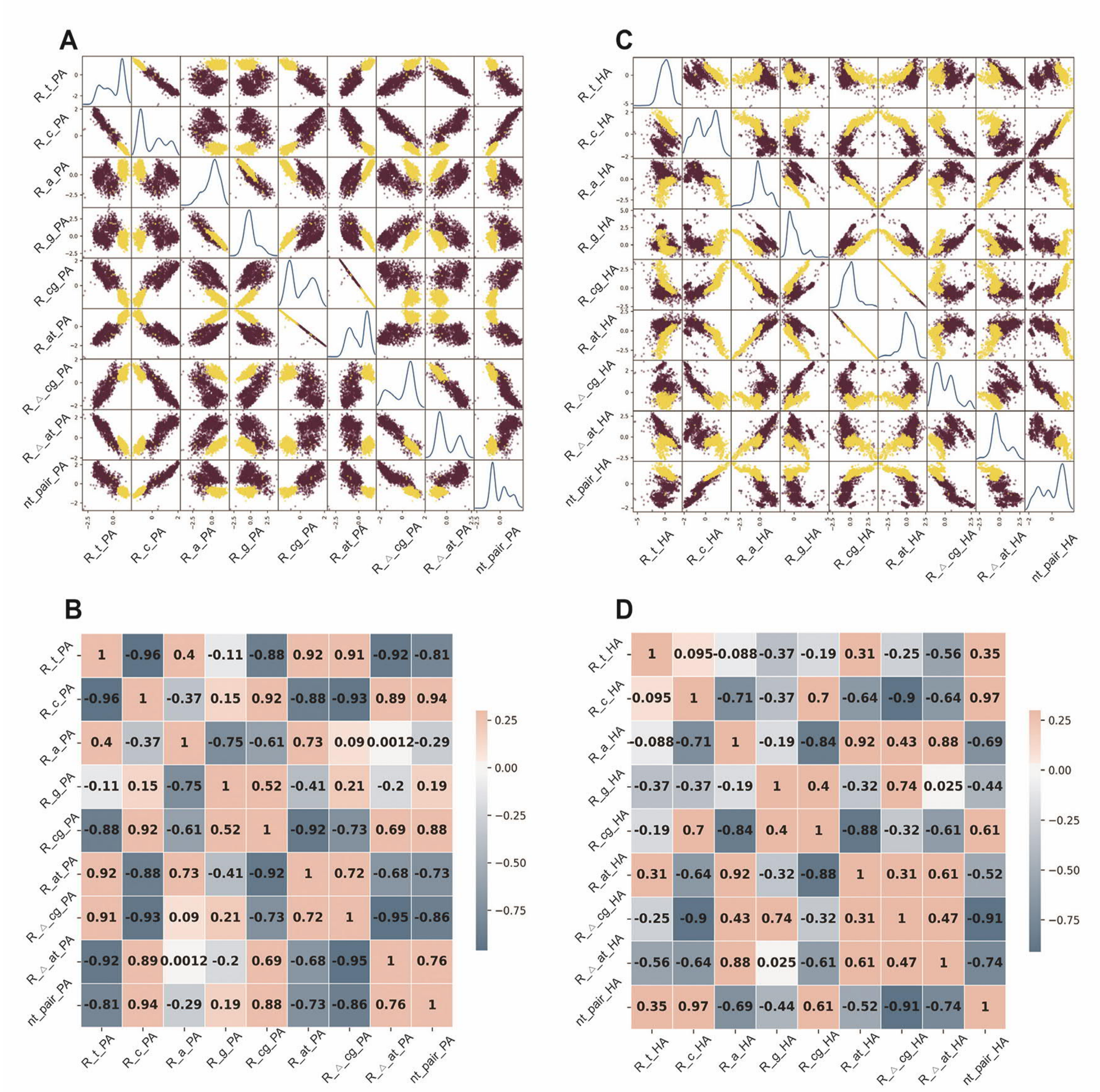

Suppl Figure 8

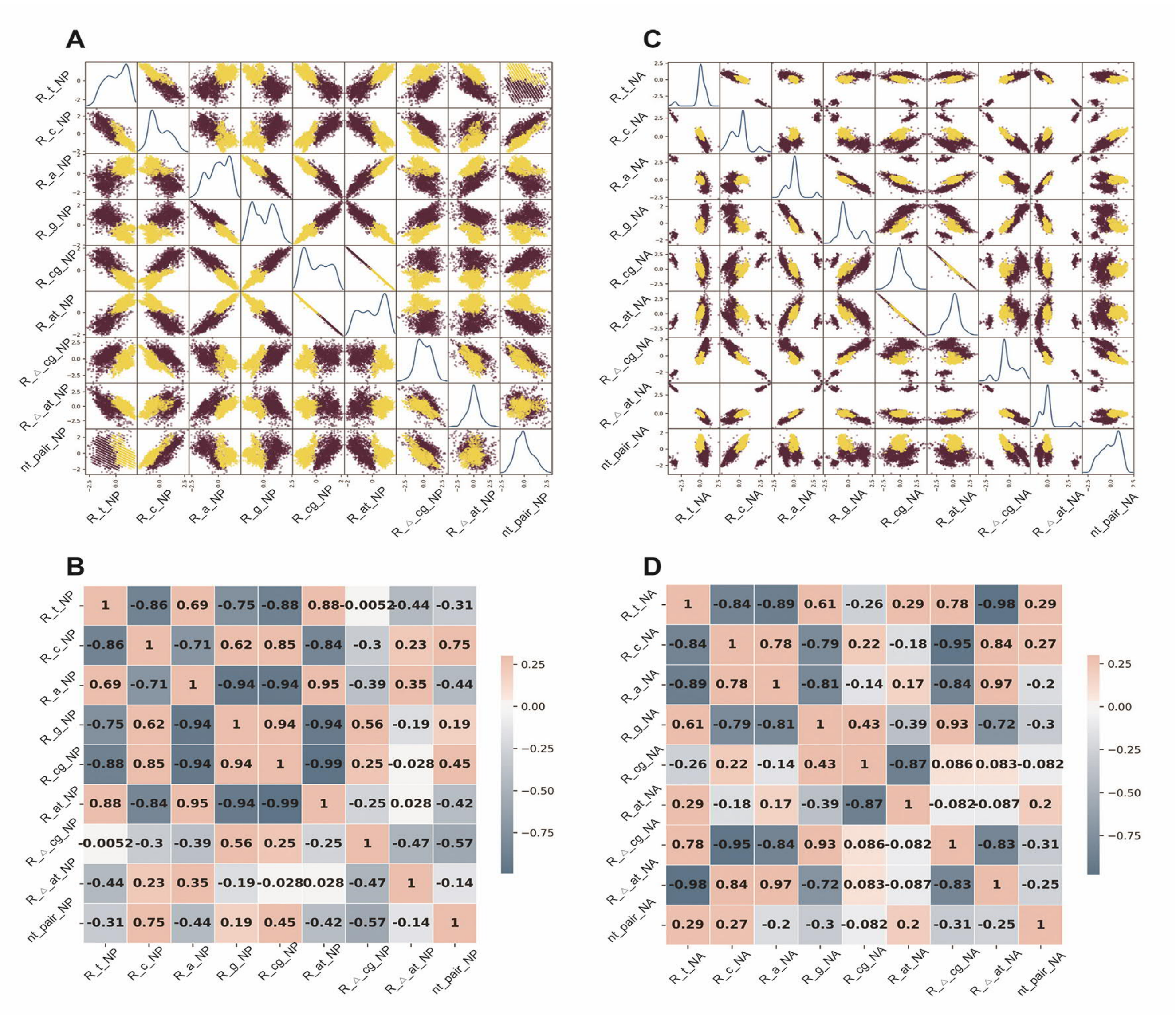

Suppl Figure 9

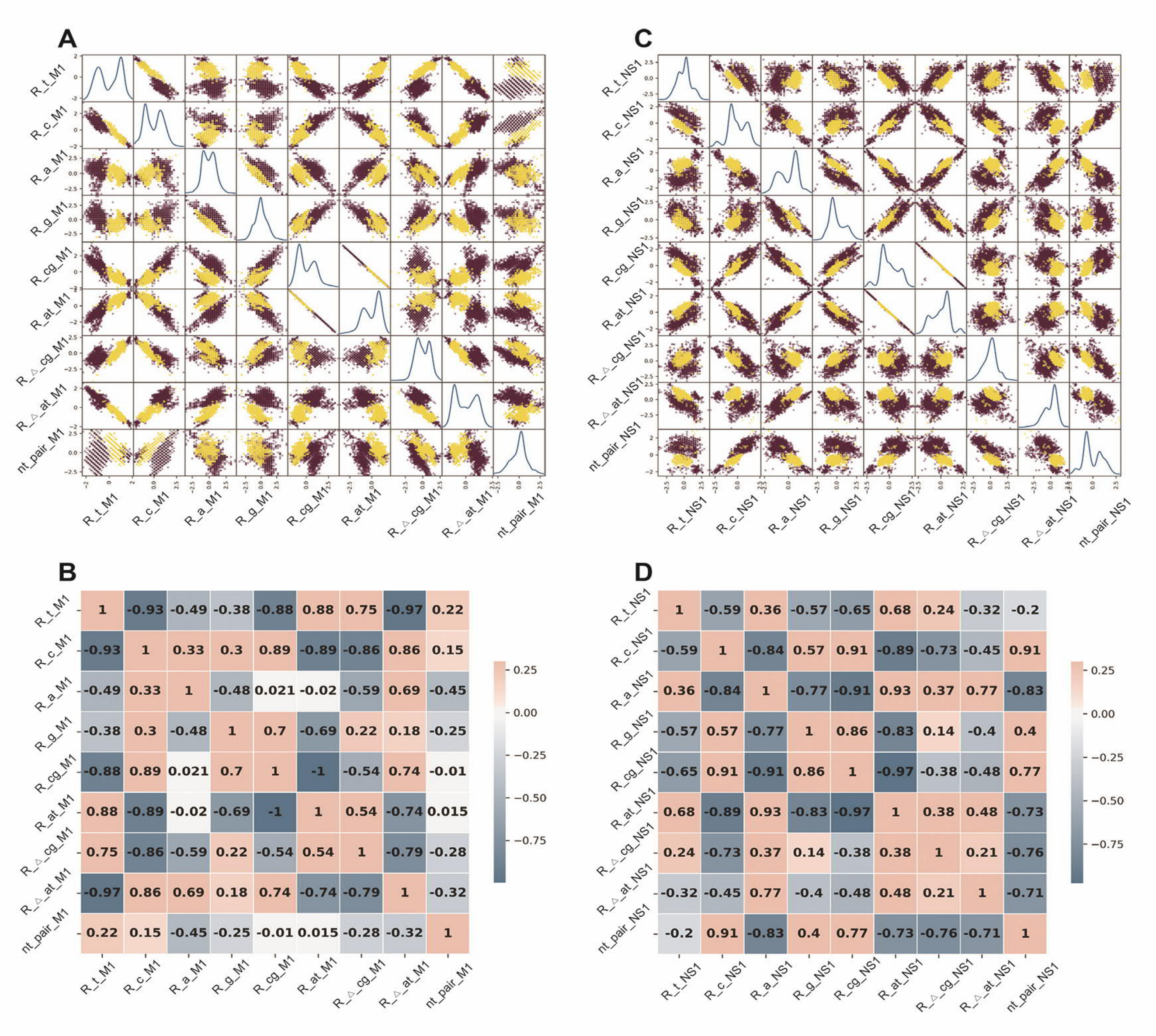

Suppl Figure 10

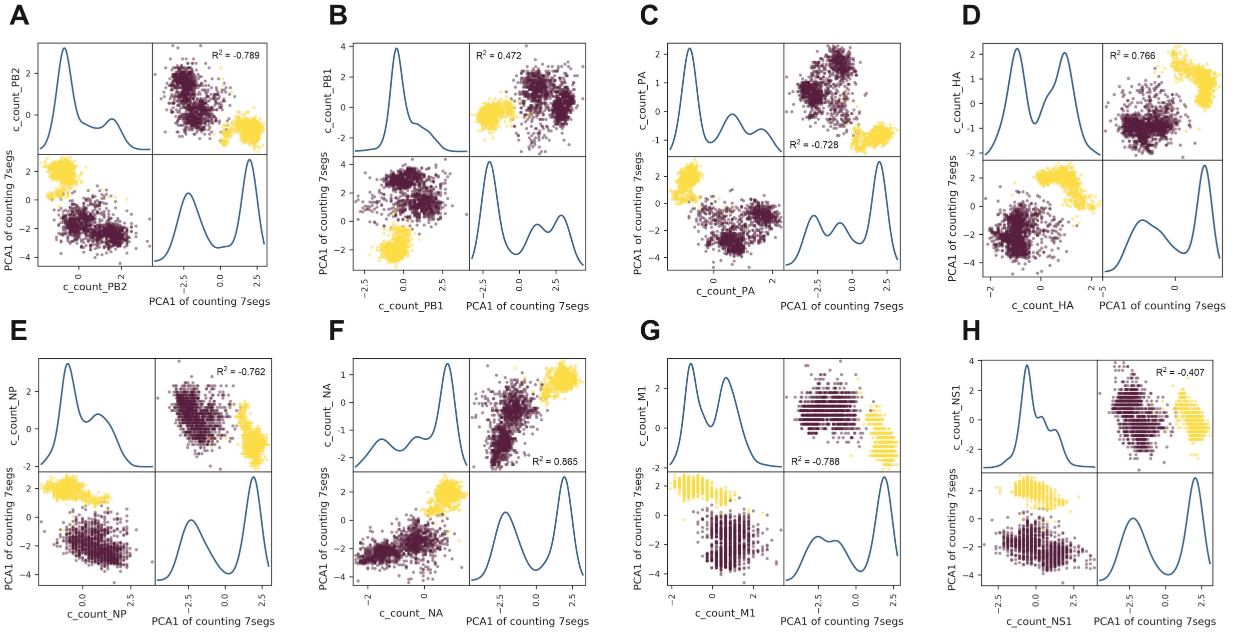

Suppl Figure 11

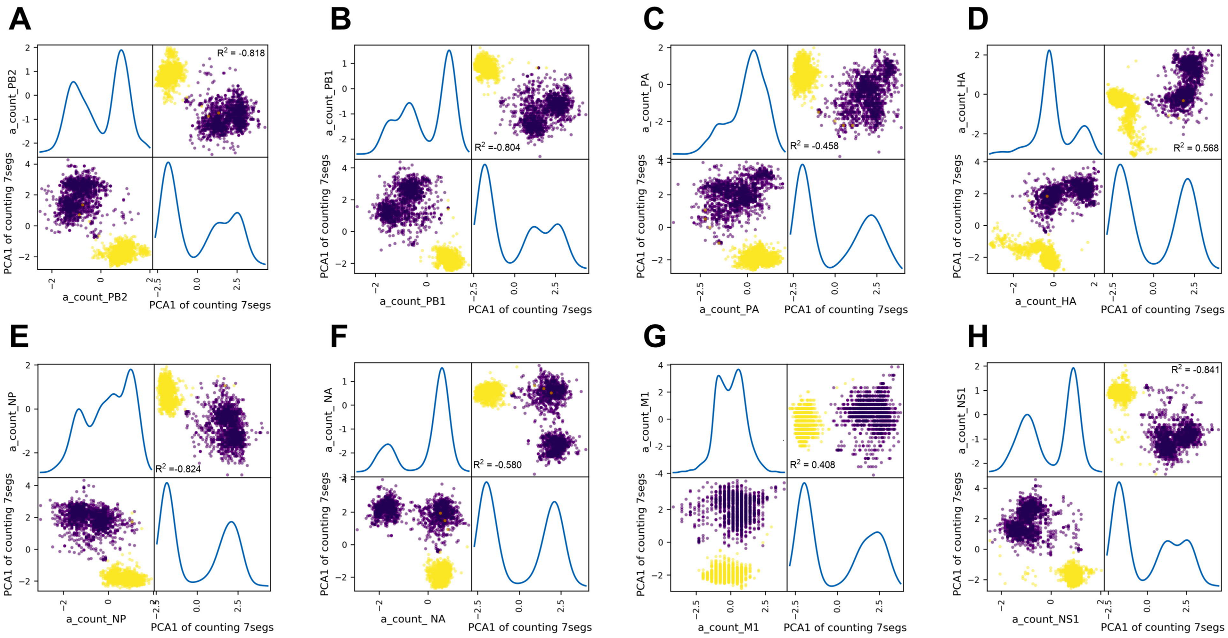

Suppl Figure 12

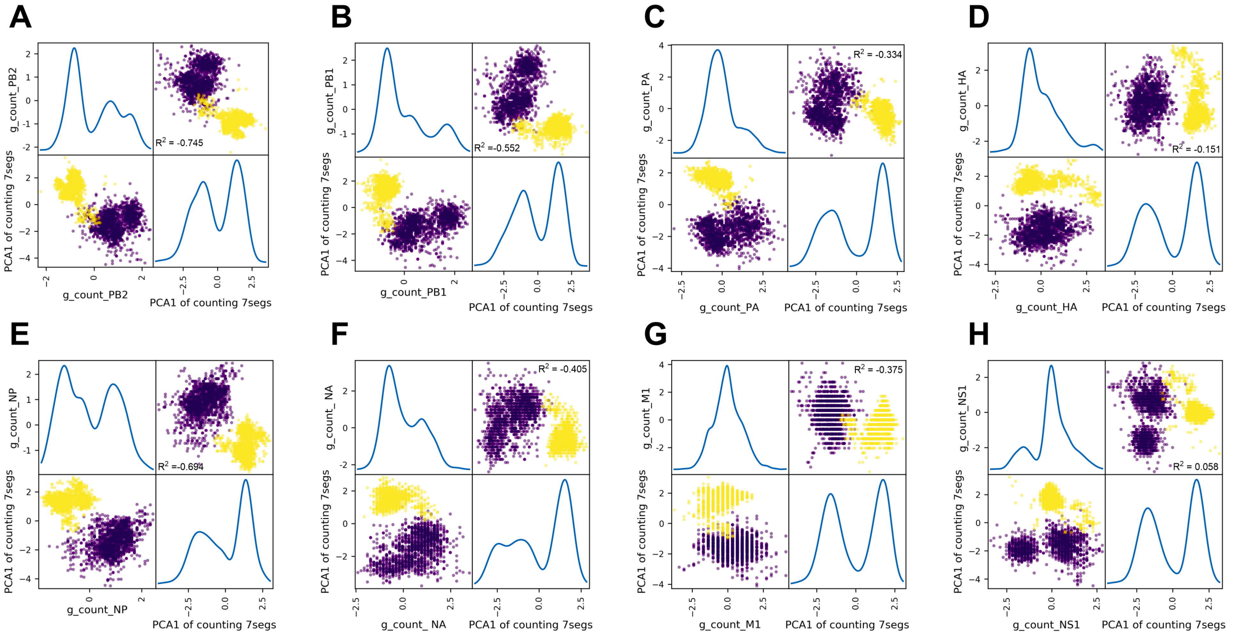

Suppl Figure 13

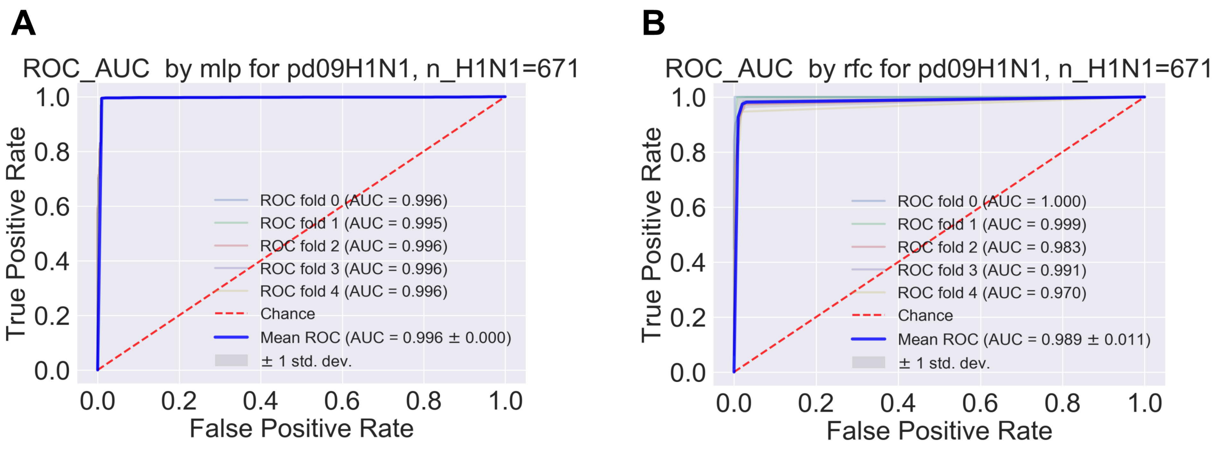

Suppl Figure 14

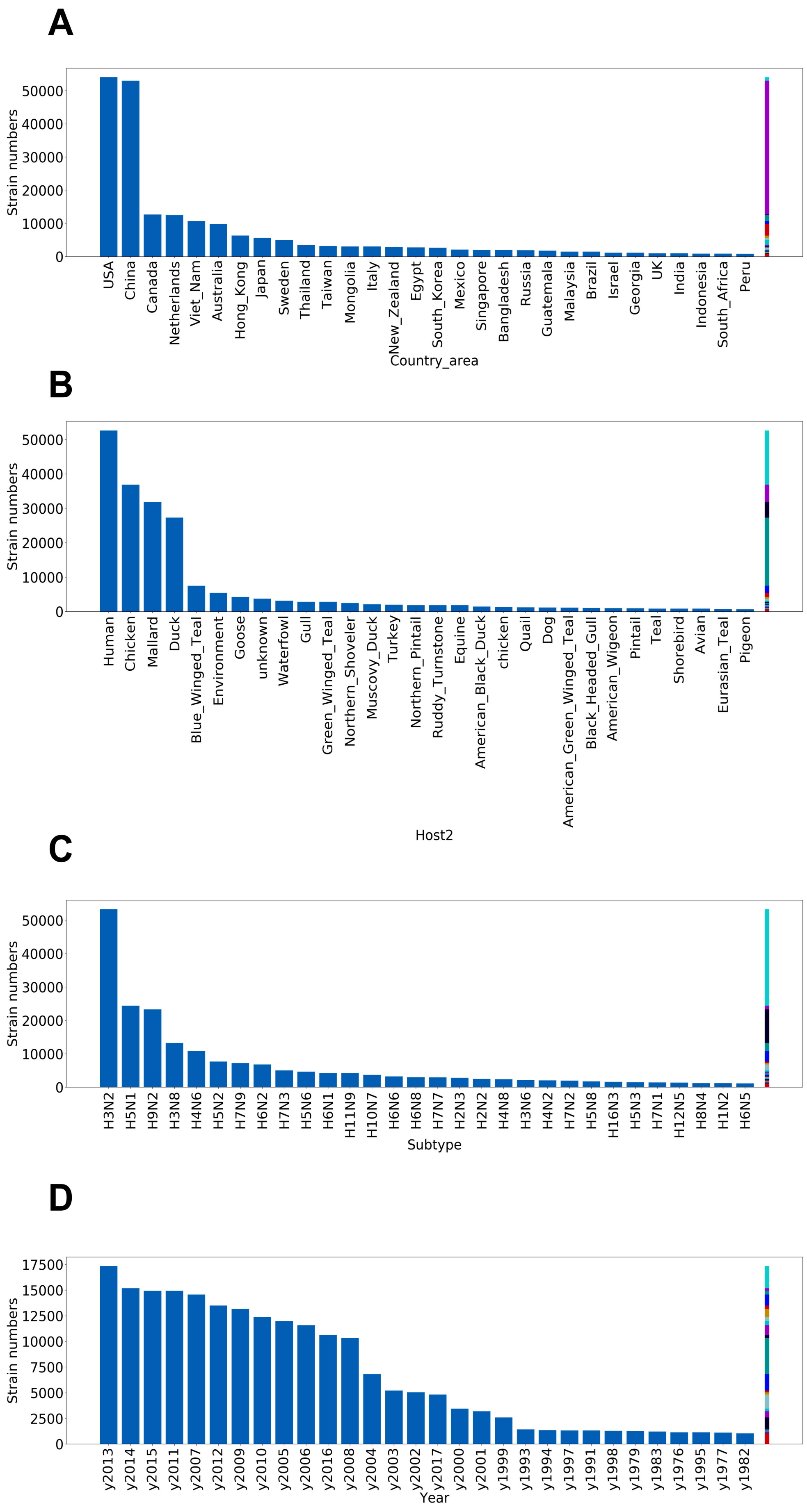

Suppl Figure 15

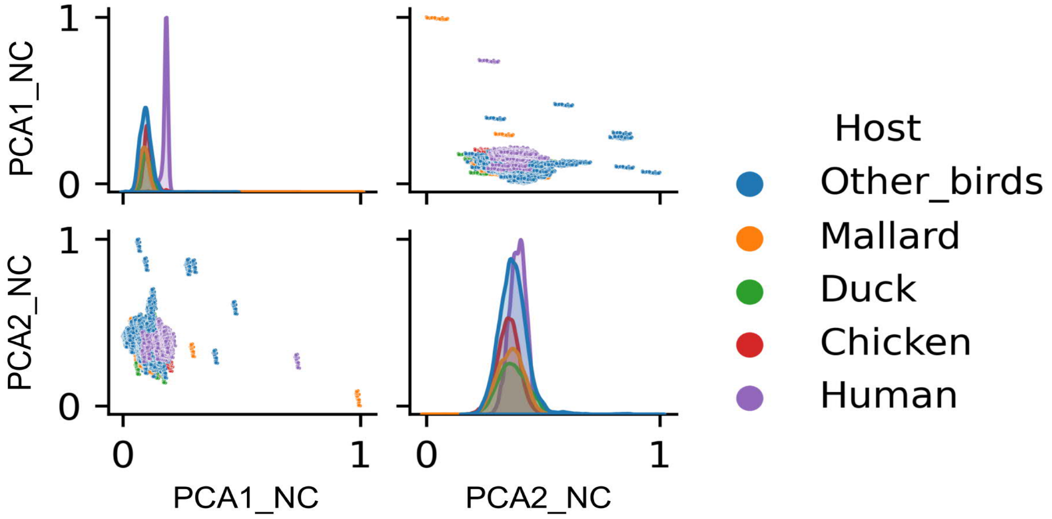

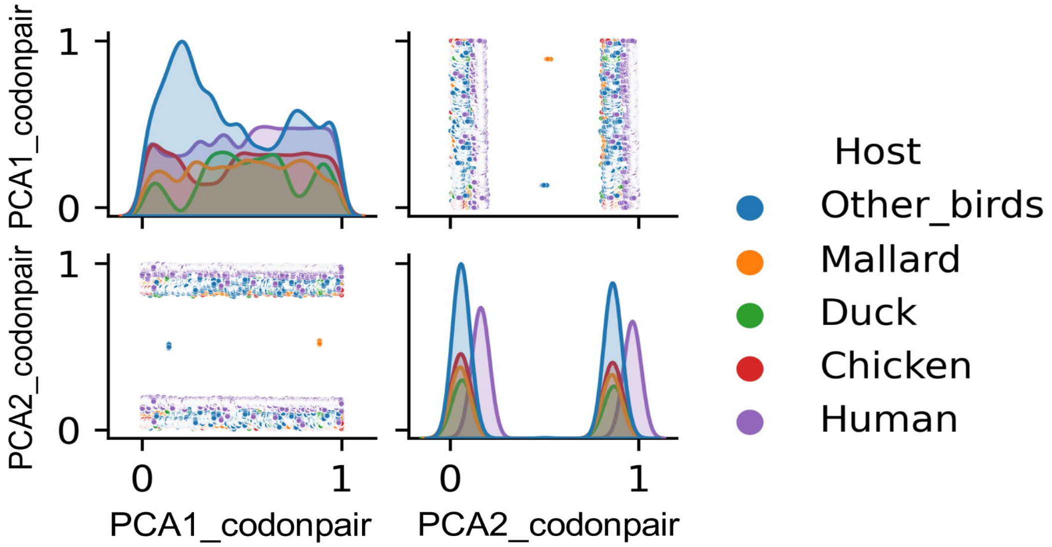

Supplement: Supplementary file 1 [file Data_Sheet_1.pdf]
