# Supplementary material for: Machine learning methods for predicting human-adaptive influenza A virus reassortment based on intersegment constraint
Source: Front Microbiol. 2025 Mar 21;16:1546536. doi: 10.3389/fmicb.2025.1546536 (PMC11970406; doi:10.3389/fmicb.2025.1546536)
Supplement: Supplementary file 2 [file Data_Sheet_2.docx]

**Supplementary Materials for**

**Machine Learning Methods for Predicting Human-adaptive Influenza A Virus Reassortment Based on inter-segment constraint**

Running title:

**Inter-segment nt Constrains on IAV Reassortment**

Dan-Dan Zeng ^1,2†^, Yu-Rong Cai ^2†^, Sen Zhang ^2^, Fang Yan^1^*, Tao Jiang ^2^*, Jing Li ^2^*

1. College of Veterinary Medicine, Shanxi Agricultural University, Taigu, Jinzhong, 030801, China
2. State Key Laboratory of Pathogen and Biosecurity, Academy of Military Medical Sciences, Beijing, 100071, China

†These authors contributed equally to this work.

* Correspondence:

([lj-pbs@163.com](mailto:lj-pbs@163.com)) Jing Li, ([yanfang6615@163.com](mailto:yanfang6615@163.com)) Fang Yan, [jiangtao@bmi.ac.cn](mailto:jiangtao@bmi.ac.cn) (Tao Jiang)

**Supplementary results**

1. **Supplementary Tables**

**Supplementary table 1. Statistical analysis of nucleotide composition features between avian and mammalian IAVs.**

Statistical analysis for the difference between avian and mammalian sequence samples in each of the nucleotide composition features was performed with the Mann-Whitney U test by scipy.stats.mannwhitneyu model (SciPy, Python).

| **Nucleotide**  **composition** | **p-value of t-test between avian and mammalian sequences** | | | | | | | | |
| --- | --- | --- | --- | --- | --- | --- | --- | --- | --- |
|  | **strain** | **PB2** | **PB1** | **PA** | **HA** | **NP** | **NA** | **M1** | **NS1** |
| Ratio_t | <0.001 | <0.001 | <0.001 | <0.001 | <0.001 | <0.001 | <0.001 | <0.001 | <0.001 |
| Ratio_c | <0.001 | <0.001 | <0.001 | <0.001 | <0.001 | <0.001 | <0.001 | <0.001 | <0.001 |
| Ratio_a | <0.001 | <0.001 | <0.001 | <0.001 | <0.001 | <0.001 | <0.001 | <0.001 | <0.001 |
| Ratio_g | <0.001 | <0.001 | <0.001 | <0.001 | <0.001 | <0.001 | <0.001 | <0.001 | <0.001 |
| Ratio_cg | <0.001 | <0.001 | <0.001 | <0.001 | <0.001 | <0.001 | <0.001 | <0.001 | <0.001 |
| Ratio_at | <0.001 | <0.001 | <0.001 | <0.001 | <0.001 | <0.001 | <0.001 | <0.001 | <0.001 |
| Ratio_delta_cg | <0.001 | <0.001 | <0.001 | <0.001 | <0.001 | <0.001 | <0.001 | <0.001 | <0.001 |
| Ratio_delta_at | <0.001 | <0.001 | <0.001 | <0.001 | <0.001 | <0.001 | <0.001 | <0.001 | <0.001 |
| nt_pair | 0.618 | <0.001 | <0.001 | <0.001 | <0.001 | <0.001 | <0.001 | <0.001 | <0.001 |

**Supplementary table 2. Statistical analysis of nucleotide composition features between IAVs and IBVs.**

Statistical analysis for the difference between avian and mammalian sequence samples in each of the nucleotide composition features was performed with the Mann-Whitney U test by scipy.stats.mannwhitneyu model (SciPy, Python).

| **Nucleotide**  **composition** | **p-value of t-test between avian and mammalian sequences** | | | | | | | | |
| --- | --- | --- | --- | --- | --- | --- | --- | --- | --- |
|  | **strain** | **PB2** | **PB1** | **PA** | **HA** | **NP** | **NA** | **M1** | **NS1** |
| Ratio_t | <0.001 | <0.001 | <0.001 | <0.001 | <0.001 | <0.001 | <0.001 | <0.001 | <0.001 |
| Ratio_c | <0.001 | <0.001 | <0.001 | <0.001 | <0.001 | <0.001 | <0.001 | <0.001 | <0.001 |
| Ratio_a | <0.001 | <0.001 | <0.001 | <0.001 | <0.001 | <0.001 | <0.001 | <0.001 | <0.001 |
| Ratio_g | <0.001 | <0.001 | <0.001 | <0.001 | <0.001 | <0.001 | <0.001 | <0.001 | <0.001 |
| Ratio_cg | <0.001 | <0.001 | <0.001 | <0.001 | <0.001 | <0.001 | <0.001 | <0.001 | <0.001 |
| Ratio_at | <0.001 | <0.001 | <0.001 | <0.001 | <0.001 | <0.001 | <0.001 | <0.001 | <0.001 |
| Ratio_delta_cg | <0.001 | <0.001 | <0.001 | <0.001 | <0.001 | <0.001 | <0.001 | <0.001 | <0.001 |
| Ratio_delta_at | <0.001 | <0.001 | <0.001 | <0.001 | <0.001 | <0.001 | <0.001 | <0.001 | <0.001 |
| nt_pair | <0.001 | <0.001 | <0.001 | <0.001 | <0.001 | <0.001 | <0.001 | <0.001 | <0.001 |

**Supplementary table 3.**

**Adaptation ratio (adapted / total) of simulated reassortants between pd09H1N1 EPplus and the IAV of varied country / areas.**

| Country_area | Duck | Mallard | Chicken | Other_birds | Human | Score |
| --- | --- | --- | --- | --- | --- | --- |
| Egypt | 0.544444 |  | 0.562121 | 0.127778 | 1 | 0.336111 |
| South_Korea | 0 | 0.5 | 0.338889 | 0.263593 | 1 | 0.301241 |
| Viet_Nam | 0.256579 | 0.196429 | 0.154401 | 0.271991 | 1 | 0.226504 |
| Australia | 0.333333 |  | 0 | 0.386574 | 1 | 0.166667 |
| Canada | 0.154762 | 0.07186 | 0.155556 | 0.068233 | 0.948413 | 0.113311 |
| USA | 0.206019 | 0.108291 | 0.080882 | 0.11327 | 1 | 0.11078 |
| Russia | 0.194444 | 0 | 0.194444 | 0.002778 | 1 | 0.098611 |
| Guatemala | |  | 0.75 | 0.179977 |  | 0.089988 |
| China | 0.266107 | 0.001263 | 0.053419 | 0.121407 | 0.890572 | 0.087413 |
| Taiwan | 0.070513 |  | 0.077423 | 0.268519 | 1 | 0.073968 |
| Bangladesh | 0.055556 | 0 | 0.196759 | 0.076389 |  | 0.065972 |
| Japan | 0.04 | 0 | 0.059829 | 0.256173 | 0.998737 | 0.049915 |
| Mexico |  |  | 0.286616 | 0.017361 | 0.952107 | 0.008681 |
| Hong_Kong | 0 | 0 | 0.042146 | 0.011628 | 0.999346 | 0.005814 |
| Mongolia | 0 | 0 |  | 0.019157 |  | 0 |
| India | 0 |  | 0.061404 | 0 | 1 | 0 |
| Pakistan |  |  | 0.024074 | 0 |  | 0 |
| Laos | 0 |  | 0 |  |  | 0 |
| Chile |  |  |  | 0.034188 | 1 | 0 |
| Nicaragua |  |  |  |  | 1 | 0 |
| France | 0 | 0 | 0 | 0.25 | 1 | 0 |
| Germany | 0 | 0 | 0 | 0 | 1 | 0 |
| Peru |  |  |  | 1 | 1 | 0 |
| Indonesia | 0 |  | 0 | 0 | 1 | 0 |
| South_Africa | 0 |  |  | 0.058824 | 1 | 0 |
| Georgia | 0 | 0 |  | 0.270202 |  | 0 |
| UK | 0 | 0 | 0 | 0.5 | 1 | 0 |
| Italy | 0 | 0 | 0 | 0.102564 | 0.966667 | 0 |
| Israel |  |  | 0.001111 | 0 |  | 0 |
| Brazil |  |  |  | 0.666667 | 1 | 0 |
| Malaysia | 0 |  |  |  | 1 | 0 |
| Netherlands | 0 | 0 | 0 | 0.320222 | 0.999681 | 0 |
| Singapore |  |  |  |  | 1 | 0 |
| Sweden |  | 0 |  | 0 | 1 | 0 |
| Thailand | 0 |  | 0 | 0 | 1 | 0 |
| New_Zealand | 0.611111 | 0 |  |  | 1 | 0 |
| Cambodia | 0.013889 |  | 0 | 0 | 1 | 0 |

1. **Supplementary Figures**

**Supplementary Figure 1. Distribution in the labels of Country_area, Host, Subtypes and Year of influenza A virus (IAV) strain samples, up to Dec 31^st^, 2018.**

Labels of Country_area (A), Host (B), Subtype (C) and Year (D) for 12,400 IAV strains with full eight segment coding sequences were counted and plotted. Values were sorted with a descending turn, and the y-axis was set with linear tick, with values sorted with a descending turn.

**Supplementary Figure 2. Distribution in Country_area, Host, Subtypes, or Year of IAV strain samples, post resampling.**

A stochastic resampling was performed to reduce the distribution bias on the USA of the Country_area label and on post-2009 of the Year label. The rest 9, 525 strains from different countries/areas (A), hosts (B), subtypes (C) and years (D) were counted and plotted, with values sorted with a descending turn.

**Supplementary Figure 3. Violinplot of the nucleotide composition factors for influenza A and B viruses (IAVs, IBVs).**

The frequency of nucleotide T, C, A or G (R_t, c, a or g_), the frequency of GC or AT content (R_at_ or R_cg_), the relative levels of nucleotide bias (R_Δ_at_ or R_Δ_cg_) and of nucleotide pair (nt_pair_) were counted strain-dependently or segment-dependently (PB2, PB1,PA, HA, NP, NA, M1 or NS1) respectively (A-I); relative frequency value was plotted with Violinplot (seaborn model, Python), IAVs and IBVs were respectively plotted in color of blue and orange. Data were standardized as (value - value mean) / value std. A p-value for each factor was indicated independently.

**Supplementary Figure 4. Hierarchical clustering of human and avian IAV sequences based on the Euclidean distance of nucleotide composition features.**

Approximately 60 IAV strain samples were randomly (random state = 1) selected from the 9,525 IAV strain set, and then were clustered with heatmap and hierarchical clustering for PB2, PB1, PA and HA (A-D, respectively), based on the Euclidean distance of the 9 nucleotide composition features; Sequence identity and features were clustered respectively.

**Supplementary Figure 5. Hierarchical clustering of human and avian IAV sequences based on the Euclidean distance of nucleotide composition features.**

Approximately 60 IAV strain samples were randomly (random state = 1) selected from the 9,525 IAV strain set, and then were clustered with heatmap and hierarchical clustering for NP, NA, M1 and NS1 (A-D, respectively), based on the Euclidean distance of the 9 nucleotide composition features; Sequence identity and features were clustered respectively.

**Supplementary Figure 6. Correlation analysis of nucleotide composition features for the PB2 and PB1 of human and avian IAV strains.**

Every two nucleotide composition features were plotted in pair (A and C), with the pandas.scatter_matrix, with human samples plotting in yellow and avian samples in brown, and with the sample distribution in each feature plotted by kernel density estimation (KDE). Correlation between every two features were calculated by the pandas.corr(‘Pearson’) model (Python) (B and D). 0.3 and -0.3 were set as the threshold of R^2^ respectively for positive and negative correlation.

**Supplementary Figure 7. Correlation analysis of nucleotide composition features for the PA and HA of human and avian IAV strains.**

Every two nucleotide composition features were plotted in pair (A and C), with the pandas.scatter_matrix, with human samples plotting in yellow and avian samples in brown, and with the sample distribution in each feature plotted by kernel density estimation (KDE). Correlation between every two features were calculated by the pandas.corr(‘Pearson’) model (Python) (B and D). 0.3 and -0.3 were set as the threshold of R^2^ respectively for positive and negative correlation.

**Supplementary Figure 8. Correlation analysis of nucleotide composition features for the NP and NA of human and avian IAV strains.**

Every two nucleotide composition features were plotted in pair (A and C), with the pandas.scatter_matrix, with human samples plotting in yellow and avian samples in brown, and with the sample distribution in each feature plotted by kernel density estimation (KDE). Correlation between every two features were calculated by the pandas.corr(‘Pearson’) model (Python) (B and D). 0.3 and -0.3 were set as the threshold of R^2^ respectively for positive and negative correlation.

**Supplementary Figure 9. Correlation analysis of nucleotide composition features for the M1 and NS1 of human and avian IAV strains.**

Every two nucleotide composition features were plotted in pair (A and C), with the pandas.scatter_matrix, with human samples plotting in yellow and avian samples in brown, and with the sample distribution in each feature plotted by kernel density estimation (KDE). Correlation between every two features were calculated by the pandas.corr(‘Pearson’) model (Python) (B and D). 0.3 and -0.3 were set as the threshold of R^2^ respectively for positive and negative correlation.

**Supplementary Figure 10. Principal component analysis (PCA) of nucleotide Cytosine composition between each segment and the other seven segments for IAVs.**

Cytosine composition (Ratio_c_) for every seven segments (A-H for PB2 and the other seven segments) were converted into one principal component (PCA model from sklearn.decomposition), along with Ratio_c_PB2, were scattered with scatter_matrix (pandas.plotting, Python). The correlation of the Ratio_c between each segment and the PCA1 of other seven segments or the correlation of the two PCA1 for both groups of segments were analyzed with Pearson correlation model of Pandas (Python) and were indicated as R^2^ respectively. Data of nucleotide ratio were standardized as (value - value mean) / value std. 0.3 and -0.3 were set as the threshold of R^2^ respectively for positive and negative correlation.

**Supplementary Figure 11. Principal component analysis (PCA) of nucleotide Adenine composition between each segment and the other seven segments for IAVs.**

Adenine composition (Ratio_a_) for every seven segments (A-H for PB2 and the other seven segments) were converted into one principal component (PCA model from sklearn.decomposition), along with Ratio_a_PB2, were scattered with scatter_matrix (pandas.plotting, Python). The correlation of the Ratio_a between each segment and the PCA1 of other seven segments or the correlation of the two PCA1 for both groups of segments were analyzed with Pearson correlation model of Pandas (Python) and were indicated as R^2^ respectively. Data of nucleotide ratio were standardized as (value - value mean) / value std. 0.3 and -0.3 were set as the threshold of R^2^ respectively for positive and negative correlation.

**Supplementary Figure 12. Principal component analysis (PCA) of nucleotide Guanine composition between each segment and the other seven segments for IAVs.**

Cytosine composition (Ratio_g_) for every seven segments (A-H for PB2 and the other seven segments) were converted into one principal component (PCA model from sklearn.decomposition), along with Ratio_g_PB2, were scattered with scatter_matrix (pandas.plotting, Python). The correlation of the Ratio_g between each segment and the PCA1 of other seven segments or the correlation of the two PCA1 for both groups of segments were analyzed with Pearson correlation model of Pandas (Python) and were indicated as R^2^ respectively. Data of nucleotide ratio were standardized as (value - value mean) / value std. 0.3 and -0.3 were set as the threshold of R^2^ respectively for positive and negative correlation.

**Figure 13. Prediction of the simulated reassortant pd2009H1N1 viruses with the H1N1 IAVs post 2009.**

Human adaption (True/False) of the simulated reassortant pd2009H1N1 viruses with the H1N1 IAVs post 2009 were predicted by mlp (A) and rfc (B) model. The Receiver Operating Characteristic (ROC) and Area Under ROC Curve (AUC) for the rfc model with the nine nucleotide composition features were indicated respectively for both models. The sample number was noted in each subplot. Training data were randomly split into five folds; 1x Standard deviation ( ± 1 std.dev) was adopted for ROC and AUC. The human adaptation was predicted by the rfc model with a probability threshold of 0.5.

**Supplementary Figure 14. Distribution in Country_area, Host, Subtypes, or Year of the simulated reassortant pd2009H1N1 viruses.**

Labels of Country_area (A), Host (B), Subtype (C) and Year (D) for 221,184 simulated viruses with full eight segment coding sequences were counted and plotted. Values were sorted with a descending turn, and the y-axis was set with linear tick, with values sorted with a descending turn.

**Supplementary Figure 15. Pairplot of PCA-reduced genomic NC or codonpair traits of the IAVs from various hosts.**

Genomic NC (A) or codonpair (B) features were reduced into two components by PCA method and were visualized by pairplot, with virus host labeled in color.
